# Supplementary material for: Delving into female breast cancer: Distinct disease-specific survival outcomes between invasive lobular and ductal carcinomas revealed by propensity score matching
Source: PLoS One. 2024 Dec 23;19(12):e0300116. doi: 10.1371/journal.pone.0300116 (PMC11665987; doi:10.1371/journal.pone.0300116)
Supplement: S1 Table — (DOCX) [file pone.0300116.s001.docx]

**S1 Table. IDC group univariate and multivariate Cox regression analysis.**

| **IDC uniCOX(N=58497)** | | | | | |  | **IDC multiCOX(N=58497)** | |
| --- | --- | --- | --- | --- | --- | --- | --- | --- |
| **Characteristic** | | **No.** | **HR（95%CI）** | **P** | **global.pval** |  | **HR（95%CI）** | **P** |
| age | |  |  |  |  |  |  |  |
|  | <60 | 28806 | 1.00[Reference] |  | <0.001 |  | 1.00[Reference] |  |
|  | >=60 | 29691 | 1.16(1.10-1.22) | <0.001 |  |  | 1.36(1.29-1.44) | <0.001 |
| race | |  |  |  |  |  |  |  |
|  | White | 48103 | 1.00[Reference] |  |  |  | 1.00[Reference] |  |
|  | Black | 5339 | 2.00(1.87-2.15) | <0.001 | <0.001 |  | 1.23(1.14-1.33) | <0.001 |
|  | Other | 5055 | 0.70(0.63-0.78) | <0.001 |  |  | 0.78(0.69-0.87) | <0.001 |
| Maritial status | |  |  |  |  |  |  |  |
|  | Married | 35952 | 1.00[Reference] |  |  |  | 1.00[Reference] |  |
|  | Divorced | 6880 | 1.41(1.30-1.53) | <0.001 | <0.001 |  | 1.15(1.06-1.25) | 0.001 |
|  | Widowed | 6299 | 2.42(2.26-2.60) | <0.001 |  |  | 1.84(1.71-1.98) | <0.001 |
|  | single | 9366 | 1.64(1.53-1.76) | <0.001 |  |  | 1.21(1.13-1.30) | <0.001 |
| Primary Site | |  |  |  |  |  |  |  |
|  | Central | 2376 | 1.00[Reference] |  | <0.001 |  | 1.00[Reference] |  |
|  | Upper inner | 7869 | 0.62(0.54-0.71) | <0.001 |  |  | 0.96(0.84-1.11) | 0.605 |
|  | Lower inner | 3466 | 0.69(0.59-0.81) | <0.001 |  |  | 1.07(0.91-1.25) | 0.437 |
|  | Upper outer | 20256 | 0.70(0.62-0.79) | <0.001 |  |  | 0.89(0.79-1.01) | 0.061 |
|  | Lower outer | 4659 | 0.76(0.66-0.88) | <0.001 |  |  | 1.01(0.87-1.16) | 0.947 |
|  | Overlapping | 13621 | 0.72(0.63-0.81) | <0.001 |  |  | 0.93(0.82-1.06) | 0.266 |
|  | others | 6250 | 1.32(1.16-1.50) | <0.001 |  |  | 1.11(0.98-1.27) | 0.105 |
| Subtype | |  |  |  |  |  |  |  |
|  | HR+/HER2+ | 6687 | 1.00[Reference] |  |  |  | 1.00[Reference] |  |
|  | HR+/HER2- | 42081 | 0.74(0.68-0.80) | <0.001 | <0.001 |  | 1.48(1.36-1.62) | <0.001 |
|  | HR-/HER2+ | 2807 | 1.49(1.31-1.68) | <0.001 |  |  | 0.46(0.36-0.57) | <0.001 |
|  | HR-/HER2- | 6922 | 2.33(2.13-2.55) | <0.001 |  |  | 1.04(0.84-1.28) | 0.743 |
| Grade | |  |  |  |  |  |  |  |
|  | I | 13678 | 1.00[Reference] |  |  |  | 1.00[Reference] |  |
|  | II | 24247 | 3.15(2.80-3.53) | <0.001 | <0.001 |  | 1.76(1.56-1.98) | <0.001 |
|  | III | 20494 | 7.56(6.76-8.45) | <0.001 |  |  | 2.66(2.36-3.01) | <0.001 |
|  | IV | 78 | 11.60(7.38-18.2) | <0.001 |  |  | 4.95(3.15-7.79) | <0.001 |
| Summary stage | |  |  |  |  |  |  |  |
|  | Localized | 39655 | 1.00[Reference] |  |  |  | 1.00[Reference] |  |
|  | Regional | 16713 | 3.91(3.68-4.16) | <0.001 | <0.001 |  | 1.69(1.54-1.84) | <0.001 |
|  | Distant | 2129 | 28.40(26.50-30.50) | <0.0001 |  |  | 2.85(2.07-3.93) | <0.001 |
| AJCC | |  |  |  |  |  |  |  |
|  | I | 32596 | 1.00[Reference] |  |  |  | 1.00[Reference] |  |
|  | II | 18585 | 4.03(3.72-4.36) | <0.001 | <0.001 |  | 1.79(1.58-2.03) | <0.001 |
|  | III | 5280 | 12.00(11.10-13.10) | <0.001 |  |  | 3.84(3.30-4.47) | <0.001 |
|  | IV | 2036 | 45.80(42.10-49.80) | <0.001 |  |  | 6.49(4.60-9.16) | <0.001 |
| Laterality | |  |  |  |  |  |  |  |
|  | Right | 29037 | 1.00[Reference] |  | 0.747 |  | NA |  |
|  | Left | 29460 | 1.01(0.96-1.06) | 0.747 |  |  | NA(NA) | NA |
| Systemic Sur Seq | |  |  |  |  |  |  |  |
|  | AAT | 39718 | 1.00[Reference] |  |  |  | 1.00[Reference] |  |
|  | NSOST | 11795 | 3.11(2.93-3.30) | <0.001 | <0.001 |  | 2.25(2.10-2.40) | <0.001 |
|  | others | 6984 | 3.54(3.31-3.78) | <0.001 |  |  | 1.20(1.12-1.29) | <0.001 |
| Surg Rad Seq | |  |  |  |  |  |  |  |
|  | PORT | 33461 | 1.00[Reference] |  |  |  | 1.00[Reference] |  |
|  | NROS | 24319 | 2.24(2.12-2.36) | <0.001 | <0.001 |  | 1.52(1.43-1.61) | <0.001 |
|  | others | 717 | 1.22(0.94-1.60) | <0.001 |  |  | 1.17(0.89-1.53) | 0.260 |
| ER | |  |  |  |  |  |  |  |
|  | Positive | 48107 | 1.00[Reference] |  | <0.001 |  | 1.00[Reference] |  |
|  | Negtive | 10390 | 2.72(2.58-2.87) | <0.001 |  |  | 1.94(1.60-2.34) | <0.001 |
| PR | |  |  |  |  |  |  |  |
|  | Positive | 42019 | 1.00[Reference] |  | <0.001 |  | 1.00[Reference] |  |
|  | Negtive | 16478 | 2.68(2.54-2.82) | <0.001 |  |  | 1.64(1.52-1.77) | <0.001 |
| Tumor size | |  |  |  |  |  |  |  |
|  | <=1 | 16822 | 1.00[Reference] |  |  |  | 1.00[Reference] |  |
|  | <=2 | 21096 | 2.43(2.16-2.72) | <0.001 | <0.001 |  | 1.70(1.51-1.92) | <0.001 |
|  | <=3 | 10656 | 5.60(5.00-6.27) | <0.001 |  |  | 1.96(1.70-2.26) | <0.001 |
|  | <=4 | 4320 | 10.40(9.21-11.70) | <0.001 |  |  | 2.51(2.17-2.91) | <0.001 |
|  | <=5 | 2092 | 14.90(13.10-16.90) | <0.001 |  |  | 2.94(2.52-3.42) | <0.001 |
|  | ＞5 | 3511 | 20.80(18.60-23.30) | <0.001 |  |  | 2.76(2.39-3.19) | <0.001 |

**Abbreviation:** AAT, Adjuvant Therapy; NSOST, No systemic therapy and/or surgical therapy; PORT, Post-Operative Radiation Therapy; NROS, No radiation and/or cancer-directed surgery.
