# Supplementary material for: Delving into female breast cancer: Distinct disease-specific survival outcomes between invasive lobular and ductal carcinomas revealed by propensity score matching
Source: PLoS One. 2024 Dec 23;19(12):e0300116. doi: 10.1371/journal.pone.0300116 (PMC11665987; doi:10.1371/journal.pone.0300116)
Supplement: S2 Table — (DOCX) [file pone.0300116.s002.docx]

**S2 Table. ILC group univariate and multivariate Cox regressionanalysis.**

| ILC uniCOX(N=7908) | | | | | |  | ILC multiCOX(N=7908) | |
| --- | --- | --- | --- | --- | --- | --- | --- | --- |
| **Characteristic** | | **No.** | **HR（95%CI）** | **p.value** | **global.pval** |  | **HR（95%CI）** | **p.value** |
| age | |  |  |  | <0.001 |  |  |  |
|  | <60 | 3079 | 1.00[Reference] |  |  |  | 1.00[Reference] |  |
|  | >=60 | 4829 | 1.42(1.23-1.64) | <0.001 |  |  | 1.53(1.31-1.78) | <0.001 |
| race | |  |  |  | <0.001 |  |  |  |
|  | White | 6980 | 1.00[Reference] |  |  |  | 1.00[Reference] |  |
|  | Black | 540 | 1.38(1.09-1.74) | 0.008 |  |  | 1.15(0.90-1.47) | 0.265 |
|  | Other | 388 | 0.59(0.39-0.87) | 0.008 |  |  | 0.77(0.51-1.15) | 0.194 |
| Maritial status | |  |  |  | <0.001 |  |  |  |
|  | Married | 4895 | 1.00[Reference] |  |  |  | 1.00[Reference] |  |
|  | Divorced | 927 | 1.46(1.19-1.78) | <0.001 |  |  | 1.35(1.10-1.66) | 0.004 |
|  | Widowed | 1009 | 2.02(1.70-2.42) | <0.001 |  |  | 1.51(1.25-1.82) | <0.001 |
|  | single | 1077 | 1.35(1.11-1.65) | 0.003 |  |  | 1.08(0.88-1.32) | 0.490 |
| Primary Site | |  |  |  | <0.001 |  |  |  |
|  | Central | 379 | 1.00[Reference] |  |  |  | 1.00[Reference] |  |
|  | Upper inner | 835 | 0.41(0.29-0.59) | <0.001 |  |  | 0.73(0.51-1.06) | 0.096 |
|  | Lower inner | 321 | 0.54(0.35-0.84) | 0.006 |  |  | 0.88(0.56-1.38) | 0.574 |
|  | Upper outer | 2778 | 0.52(0.39-0.69) | <0.001 |  |  | 0.80(0.60-1.06) | 0.121 |
|  | Lower outer | 611 | 0.48(0.33-0.70) | <0.001 |  |  | 0.76(0.52-1.11) | 0.152 |
|  | Overlapping | 1832 | 0.71(0.53-0.94) | 0.017 |  |  | 0.97(0.73-1.30) | 0.849 |
|  | others | 1152 | 1.18(0.88-1.57) | 0.263 |  |  | 1.16(0.87-1.55) | 0.320 |
| Subtype | |  |  |  | <0.001 |  |  |  |
|  | HR+/HER2+ | 333 | 1.00[Reference] |  |  |  | 1.00[Reference] |  |
|  | HR+/HER2- | 7432 | 0.78(0.57-1.07) | 0.118 |  |  | 1.23(0.90-1.69) | 0.199 |
|  | HR-/HER2+ | 37 | 3.55(1.90-6.61) | <0.001 |  |  | 0.12(0.04-0.41) | <0.001 |
|  | HR-/HER2- | 106 | 3.03(1.93-4.76) | <0.001 |  |  | 0.28(0.09-0.83) | 0.021 |
| Grade | |  |  |  | <0.001 |  |  |  |
|  | I | 2495 | 1.00[Reference] |  |  |  | 1.00[Reference] |  |
|  | II | 4850 | 1.36(1.16-1.59) | <0.001 |  |  | 1.15(0.98-1.36) | 0.090 |
|  | III | 560 | 2.53(2.00-3.19) | <0.001 |  |  | 1.64(1.29-2.08) | 0.001 |
|  | IV | 3 | 0.00000771(0-Inf) | 0.984 |  |  | 0(0-Inf) | 0.986 |
| Summary stage | |  |  |  | <0.001 |  |  |  |
|  | Localized | 5052 | 1.00[Reference] |  |  |  | 1.00[Reference] |  |
|  | Regional | 2528 | 4.8(4.04-5.71) | <0.001 |  |  | 1.78(1.39-2.30) | <0.001 |
|  | Distant | 328 | 40.00(33-48.50) | <0.001 |  |  | 11.01(5.06-23.96) | <0.001 |
| AJCC | |  |  |  | <0.001 |  |  |  |
|  | I | 3500 | 1.00[Reference] |  |  |  | 1.00[Reference] |  |
|  | II | 2913 | 3.58(2.79-4.61) | <0.001 |  |  | 3.25(2.31-4.59) | <0.001 |
|  | III | 1178 | 11.90(9.32-15.20) | <0.001 |  |  | 8.18(5.42-12.33) | <0.001 |
|  | IV | 317 | 63.30(49.20-81.50) | <0.001 |  |  | 4.58(1.97-10.67) | <0.001 |
| Laterality | |  |  |  | 0.633 |  |  |  |
|  | Right | 3849 | 1.00[Reference] |  |  |  | NA |  |
|  | Left | 4059 | 1.03(0.90-1.18) | 0.633 |  |  | NA(NA-NA) | NA |
| Systemic Sur Seq | |  |  |  | <0.001 |  |  |  |
|  | AAT | 5796 | 1.00[Reference] |  |  |  | 1.00[Reference] |  |
|  | NSOST | 1489 | 2.56(2.21-2.98) | <0.001 |  |  | 2.18(1.84-2.59) | <0.001 |
|  | others | 623 | 2.91(2.40-3.54) | <0.001 |  |  | 1.26(1.02-1.56) | 0.030 |
| Surg Rad Seq | |  |  |  | <0.001 |  |  |  |
|  | PORT | 4251 | 1.00[Reference] |  |  |  | 1.00[Reference] |  |
|  | NROS | 3602 | 1.84(1.60-2.11) | <0.001 |  |  | 1.42(1.21-1.67) | <0.001 |
|  | others | 55 | 2.24(1.16-4.35) | 0.017 |  |  | 1.1(0.56-2.16) | 0.780 |
| ER | |  |  |  | <0.001 |  |  |  |
|  | Positive | 7754 | 1.00[Reference] |  |  |  | 1.00[Reference] |  |
|  | Negtive | 154 | 3.99(3.01-5.30) | <0.001 |  |  | 5.97(2.21-16.15) | <0.001 |
| PR | |  |  |  | <0.001 |  |  |  |
|  | Positive | 6606 | 1.00[Reference] |  |  |  | 1.00[Reference] |  |
|  | Negtive | 1302 | 2.36(2.03-2.73) | <0.001 |  |  | 1.95(1.66-2.29) | <0.001 |
| Tumor size | |  |  |  | <0.001 |  |  |  |
|  | <=1 | 1575 | 1.00[Reference] |  |  |  | 1.00[Reference] |  |
|  | <=2 | 2461 | 1.93(1.41-2.65) | <0.001 |  |  | 1.61(1.16-2.24) | 0.005 |
|  | <=3 | 1509 | 3.16(2.30-4.35) | <0.001 |  |  | 0.97(0.67-1.40) | 0.863 |
|  | <=4 | 680 | 5.09(3.64-7.12) | <0.001 |  |  | 1.35(0.92-1.98) | 0.121 |
|  | <=5 | 467 | 7.27(5.19-10.20) | <0.001 |  |  | 1.77(1.21-2.59) | 0.003 |
|  | ＞5 | 1216 | 8.75(6.51-11.80) | <0.001 |  |  | 1.31(0.92-1.86) | 0.131 |

**Abbreviation:** AAT, Adjuvant Therapy; NSOST, No systemic therapy and/or surgical therapy; PORT, Post-Operative Radiation Therapy; NROS, No radiation and/or cancer-directed surgery.
