# Supplementary material for: Delving into female breast cancer: Distinct disease-specific survival outcomes between invasive lobular and ductal carcinomas revealed by propensity score matching
Source: PLoS One. 2024 Dec 23;19(12):e0300116. doi: 10.1371/journal.pone.0300116 (PMC11665987; doi:10.1371/journal.pone.0300116)
Supplement: Attachment — Submitted filename: PONE-D-23-15805_R1_reviewer.pdf [file pone.0300116.s005.pdf]

PLOS ONE

Delving into Female Breast Cancer: Distinct Disease-Specific Survival outcomes Between Invasive Lobular and Ductal Carcinomas revealed by Propensity Score Matching

--Manuscript Draft--

**Manuscript Number:**	PONE-D-23-15805R1
**Article Type:**	Research Article
**Full Title:**	Delving into Female Breast Cancer: Distinct Disease-Specific Survival outcomes Between Invasive Lobular and Ductal Carcinomas revealed by Propensity Score Matching
**Short Title:**	Survival Differences Between Invasive Lobular and Ductal Carcinomas
**Corresponding Author:**	Qingxia Li<br>Hebei General Hospital<br>Shijiazhuang, CHINA
**Keywords:**	Invasive Ductal Carcinoma, Lobular Carcinomas, Prognosis, PSM
**Abstract:**	Purpose<br>The difference in prognosis between invasive lobular carcinoma (ILC) and invasive ductal carcinoma (IDC) is still controversial in the academic community. Resolving this controversy can help to more accurately determine patients' prognosis, provide further personalized treatment, alleviate unnecessary psychological burden for some patients, and provide direction for further fundamental research.<br>Patients and methods<br>A retrospective cohort study was conducted using the SEER Research Plus Data 8 Registries, Nov 2021 sub (1978-2019), including female breast cancer patients diagnosed with ILC or IDC between 2010 and 2015. Univariate and multivariate Cox regression analyses were performed, and key covariates affecting prognosis were selected. Propensity score matching (PSM) was employed to match patients, and balance tests were conducted to evaluate covariate distribution. Disease-specific survival (DSS) differences between the matched IDC and ILC groups were compared.<br>Results<br>Following PSM, the covariate differences between the IDC and ILC groups were significantly reduced. The survival analysis revealed a significantly better prognosis for the IDC group than the ILC group (Log-rank test p < 0.001), with a 28.0% increased risk observed in the ILC group.<br>Conclusion<br>This study provides evidence supporting the existence of significant differences in prognosis between IDC and ILC patients after rigorous matching. The IDC group displayed a significantly better prognosis than the ILC group. Notably, these findings have implications for personalized treatment in clinical practice and contribute to the ongoing academic debate on survival differences between IDC and ILC. However, further research is needed to investigate the biological mechanisms, gene expression, and signaling pathway disparities between IDC and ILC, aiming to provide more targeted guidance for clinical decision-making.
**Order of Authors:**	Wu Zhang
	Yuquan Huang
	Ye Zhou
	Jiaojiao Xue
	Shan Gao
	Lin Kang
	Jian Shi
	Tao Zhou
	Yalong Duan

Powered by Editorial Manager® and ProduXion Manager® from Aries Systems Corporation

	Sihan Guo
	Qingxia Li
**Response to Reviewers:**	We deeply appreciate your willingness to dedicate your valuable time to reviewing our research. Your professional expertise and precise grasp of details have provided us with invaluable and profound insights, significantly influencing the improvement of our paper. Your feedback and suggestions have not only elevated the quality of our research, but also guided us to think about the issues more comprehensively and rigorously, deepening our research. For this, we express our deep gratitude. In the process of revisiting and revising the paper, we deeply realize the importance of your review in improving our paper. We look forward to your further feedback, and believe that the revised paper will meet your expectations. Once again, we thank you for your thoughtful review and insightful suggestions. We respect and value your opinions, and we hope that our responses and changes meet your expectations.<br>Below, we have responded in detail to each review comment:<br>Reviewer 1#<br>Hi and Thanks for your Email for reviewing the article titled" Delving into Female Breast Cancer: A New Perspective on Survival Differences Between Invasive Lobular and Ductal Carcinomas Revealed by Propensity Score Matching".<br>1-This is an interesting article with novelty of the result, supporting by:<br><br>• Large scale number of cases regardless of different numbers in the clinic (>80 IDC and <10% ILC, breast cancer)<br>• Very scientific matching of both pathologic patterns, clinically and biologically and management.<br>• Novelty of result that confirm the better OS for IDC group regardless our opinion that means the same outcome or even better OS for the ILC group.<br>• Accepted the same clinical approach to care of the cases with both pathological patterns.<br><br>The author's answer： We are extremely grateful for your recognition of our research methods and results, and appreciate your professional review and feedback.<br><br>2- In my opinion there is some point of interesting that is missed during discussion and conclusion.<br>• The EMT is more expected mechanism in the ILC rather than IDC, that may be the reason of more distant metastasis.<br>• In clinic we use less adjuvant of neoadjuvant chemotherapy in ILC cases comparing to IDC.<br>• In conclusion this is accepted research with novelty with minor revision in discussion and conclusion.<br><br>The author's answer：Thank you very much for the thoughtful evaluation and valuable suggestions you provided for our research.Your observation that epithelial-mesenchymal transition (EMT) is more common in invasive lobular carcinoma (ILC) than in invasive ductal carcinoma (IDC) is indeed a critical finding. This could be due to the lack of E-cadherin, which stimulates the initiation of EMT and further leads to more common distant metastases in ILC patients. This could also be a key factor causing a poorer disease-specific survival (DSS) in ILC compared to IDC. We have added this important observation to the discussion section and hope that future basic and clinical research can explore the influence of EMT on the prognostic differences between these two pathological types and their underlying mechanisms in greater depth.<br><br>As for the influence of clinical treatment strategies on the prognostic differences between ILC and IDC you mentioned, we admit that we did not delve into it in this study. This point has been added to the limitations section of our paper. We look forward to further exploring the causal relationship between treatment methods and prognostic differences among different pathological types in future clinical research.<br><br>Once again, thank you for your guidance. We greatly value and will adopt your suggestions. We hope that through revision, the conclusions and discussions of this research will become more rigorous and comprehensive.<br><br>Reviewer #2

Powered by Editorial Manager® and ProduXion Manager® from Aries Systems Corporation

Breast cancer constitutes a crucial health concern, and investigating this disease is essential to gain deeper insights into its underlying mechanisms, thereby enhancing patient outcomes and reducing mortality rates. The availability of comprehensive and dependable data sources presents a promising avenue to tackle this challenge, and leveraging cutting-edge data analytical techniques is pivotal to expediting the analysis process and attaining more accurate results. From the title of your manuscript, it appears that your research involves adopting a new perspective on reducing bias caused by confounding variables and accessing large-scale datasets. But there are certain points that need to be addressed:

1.A similar article with the title “Comparison of Overall Survival Between Invasive Lobular Breast Carcinoma and Invasive Ductal Breast Carcinoma: A Propensity Score Matching Study Based on SEER Database” was published in “Frontiers in Oncology” in 2020 (DOI: 10.3389/fonc.2020.590643). This article utilizes the SEER dataset from 2006 to 2016 and employs the propensity score matching (PSM) method for analysis. Therefore, adopting a “new perspective” in the title of your article may not be appropriate, and your research findings could potentially serve as an update to the results presented in the aforementioned article.

The author’s answer：Thank you for your in-depth comments and for drawing our attention to the above-mentioned article. We will reconsider our title based on your suggestions.

2.On the other hand, it is advisable to refer to the aforementioned article in the introduction of your manuscript and highlight the rationale behind conducting your study, despite the existence of the previous article. In other words, explain the specific need for your research. Furthermore, in the discussion section of your research paper, it would be useful to compare your findings with those presented in the previous study.

The author’s answer：

Thank you very much for your efforts to ensure the rigor and originality of our research. You mentioned a similar article published in 2020, and we agree with your observation. We believe that although our study has similarities with this article, our research still provides important value and new insights in some key aspects. We will compare our research results with the results of this article and clarify the uniqueness and value of this study. Here are some of our differences:

Data Source: Our research data comes from the SEER Research Plus Data 8 Registries (1978-2019), while the previous study data comes from the SEER 18 tumor registry database (updated to November 2016).

Variable Coverage: Compared to the previous study, we covered a more comprehensive set of covariates for propensity score matching, including race, marital status, primary site, subtype, summary stage, tumor size, and specific types of systemic therapy (such as AAT), the specific sequence of surgery and radiotherapy, etc. These variables undoubtedly have an important impact on the prognosis of breast cancer. Compared with the variables existing in the article, we have made more detailed divisions. The inclusion of these covariates helps us understand the research topic more comprehensively and meticulously. Our Propensity Score Matching (PSM) variables are not subjectively selected, but are relatively objectively screened for variables that significantly affect prognosis through univariate and multivariate Cox regression, thereby ensuring the completeness of the included covariates and avoiding the unreliability caused by overmatching.

Method selection: Although both our study and the previous one used propensity score matching (PSM), our choice and design of statistical methods are more rigorous and detailed. To ensure the repeatability of the analysis and the reliability of the results, we provided a detailed description of the selection of variables before PSM, the specific method selection of PSM, the control of built-in parameters, and the assessment of the effect after matching (AMD and KS statistics).

Focus on Outcome: Different from the previous study, which focused on OS (Overall Survival), our research focuses on DSS (Disease-Specific Survival). This is because DSS specifically targets deaths caused by the specific disease under study and is a more accurate measure. Our choice of DSS as the main outcome helps us avoid the influence of other potential interfering factors and more accurately reflects the impact of breast cancer on survival. These factors led to our final finding that the DSS of the two groups was statistically significantly different, which might be a potential important reason for the result in that article where IDC patients showed a better OS compared to

Powered by Editorial Manager® and ProduXion Manager® from Aries Systems Corporation

ILC patients (HR=1.045, P=0.025, 95%CI: 1.007-1.085) in the comparison of OS for unmatched population database. And after matching, it turned out that there was no difference in OS between ILC and IDC patients (HR=1.017, P=0.409, 95%CI: 0.967–1.069).
We have also thoroughly considered citing the aforementioned paper in the introduction section, but we believe it would be more appropriate to cite it in the discussion section. Thus, in accordance with the reviewer's suggestion, we made detailed comparisons between our results and those of the previous study in the discussion section, and we also explained the reasons for our study. We believe this approach better demonstrates the connection between our research and other studies, and avoids giving readers the impression that our study is specifically targeting that one paper. In fact, our focus is on a class of papers exploring the controversial prognosis differences between IDC and ILC in the academic community. At this point, we would like to express our heartfelt thanks for your professional guidance. Your professional advice and rigorous academic attitude have had a significant impact on our research.

3.Given the large size of your dataset and the sufficient number of outcomes of interest, could you please provide an explanation in your manuscript that clarifies the rationale for using the propensity score method for matching the study participants based on the probability of each individual being assigned to each group?
Since the propensity score matching method failed to correctly match some variables, such as Laterality, would it not be more appropriate to use a conventional survival modeling approach, such as the Cox proportional hazard model, which adjusts for confounding variables in the regression model?

The author’s answer：

Thank you for your valuable questions. In this study, we chose the Propensity Score Matching (PSM) method primarily to reduce selection bias and deal with confounding variables. Despite our dataset's large size and the sufficient number of events, we had to deal with a lot of potential confounding variables when comparing the DSS of two groups (IDC and ILC). If we directly apply the multivariate Cox proportional hazards model, the complexity of the model might lead to unstable results. However, PSM can reduce selection bias and improve estimation bias caused by confounding variables by matching treatment and control groups in observational studies.

Regarding the issue of matching for Laterality, we didn't include it in PSM because we found no significant correlation between Laterality and DSS in the IDC and ILC groups. This is based on the univariate and multivariate Cox regression analyses we performed. In order to avoid weakening the validity and unnecessary sample loss by including too many irrelevant variables or variables not related to treatment and outcome, we chose not to include Laterality in the matching variables. We will provide additional explanations for this decision in the article.

Additionally, I completely agree with your view of the Cox proportional hazards model. In fact, in the article, we conducted KM analysis on the samples after PSM, as well as Cox proportional hazards regression. For any misunderstandings that may have been caused by a mistake in description, I deeply apologize and have made improvements and additions to the description of the Cox model results in the revised manuscript.
4.Select appropriate keywords based on Mesh in PubMed

The author’s answer：
We sincerely appreciate your comprehensive review and valuable feedback. We have made the necessary modifications to the list of keywords. The revised keywords are now better aligned with MeSH terms and accurately reflect the content of our manuscript.

5. To ensure that your reader understands the meaning of an acronym or abbreviation, provide its complete terminology when you first mention it. For example, when introducing DSS (Disease Specific Survival) in the introduction, provide its full terminology so that the reader understands what it stands for.

The author’s answer：

Powered by Editorial Manager® and ProduXion Manager® from Aries Systems Corporation

According to your advice, we have made revisions to the paper. When abbreviations or acronyms are mentioned for the first time, we have provided their complete terminology to ensure that readers can understand the meanings of the abbreviations and acronyms.

6.Please display p-values that are less than 0.001 as '< 0.001'.

The author’s answer：
Thank you very much for your suggestion. I have followed your advice and now display the results with p-values less than 0.001 as '< 0.001'.

7. Please ensure that percentages are displayed with one decimal place, and p-values are presented with three decimal places.

The author’s answer：
Thank you for your valuable suggestion. I have now adjusted the presentation to ensure that percentages are displayed with one decimal place, and p-values are presented with three decimal places, as per your recommendation. I appreciate your guidance.

Thank you once again for your valuable review and constructive feedback. It has been immensely helpful for our research. We look forward to receiving further feedback from you.

Additional Information:

**Question**	**Response**
**Financial Disclosure**<br><br>Enter a financial disclosure statement that describes the sources of funding for the work included in this submission. Review the submission guidelines for detailed requirements. View published research articles from *PLOS ONE* for specific examples.<br><br>This statement is required for submission and **will appear in the published article** if the submission is accepted. Please make sure it is accurate.	This research was supported by the Precision Medicine Joint Cultivation Program of the Natural Science Foundation of Hebei Province (No. H2022307024). Funders have no role in study design, data collection and analysis, publication decisions, or manuscript preparation.

Powered by Editorial Manager® and ProduXion Manager® from Aries Systems Corporation

**Unfunded studies**<br>Enter: *The author(s) received no specific funding for this work.*<br><br>**Funded studies**<br>Enter a statement with the following details:<br>• Initials of the authors who received each award<br>• Grant numbers awarded to each author<br>• The full name of each funder<br>• URL of each funder website<br>• Did the sponsors or funders play any role in the study design, data collection and analysis, decision to publish, or preparation of the manuscript?<br>• **NO** - Include this sentence at the end of your statement: *The funders had no role in study design, data collection and analysis, decision to publish, or preparation of the manuscript.*<br>• **YES** - Specify the role(s) played.<br><br>* typeset	
**Competing Interests**<br><br>Use the instructions below to enter a competing interest statement for this submission. On behalf of all authors, disclose any competing interests that could be perceived to bias this work—acknowledging all financial support and any other relevant financial or non-financial competing interests.<br><br>This statement is required for submission and **will appear in the published article** if the submission is accepted. Please make sure it is accurate and that any funding sources listed in your Funding Information later in the submission form are also declared in your Financial Disclosure statement.<br><br>View published research articles from *PLOS ONE* for specific examples.	The authors have declared that no competing interests exist.

Powered by Editorial Manager® and ProduXion Manager® from Aries Systems Corporation

**NO authors have competing interests**<br><br>Enter: *The authors have declared that no competing interests exist.*<br><br>**Authors with competing interests**<br><br>Enter competing interest details beginning with this statement:<br><br>*I have read the journal's policy and the authors of this manuscript have the following competing interests: [insert competing interests here]*<br><br>* typeset	
**Ethics Statement**<br><br>Enter an ethics statement for this submission. This statement is required if the study involved:<br><br>• Human participants<br>• Human specimens or tissue<br>• Vertebrate animals or cephalopods<br>• Vertebrate embryos or tissues<br>• Field research<br><br>Write "N/A" if the submission does not require an ethics statement.<br><br>General guidance is provided below. Consult the submission guidelines for detailed instructions. **Make sure that all information entered here is included in the Methods section of the manuscript.**	N/A

**Format for specific study types**<br><br>**Human Subject Research (involving human participants and/or tissue)**<br>• Give the name of the institutional review board or ethics committee that approved the study<br>• Include the approval number and/or a statement indicating approval of this research<br>• Indicate the form of consent obtained (written/oral) or the reason that consent was not obtained (e.g. the data were analyzed anonymously)<br><br>**Animal Research (involving vertebrate animals, embryos or tissues)**<br>• Provide the name of the Institutional Animal Care and Use Committee (IACUC) or other relevant ethics board that reviewed the study protocol, and indicate whether they approved this research or granted a formal waiver of ethical approval<br>• Include an approval number if one was obtained<br>• If the study involved *non-human primates*, add *additional details* about animal welfare and steps taken to ameliorate suffering<br>• If anesthesia, euthanasia, or any kind of animal sacrifice is part of the study, include briefly which substances and/or methods were applied<br><br>**Field Research**<br><br>Include the following details if this study involves the collection of plant, animal, or other materials from a natural setting:<br>• Field permit number<br>• Name of the institution or relevant body that granted permission	
**Data Availability**<br><br>Authors are required to make all data underlying the findings described fully available, without restriction, and from the time of publication. PLOS allows rare exceptions to address legal and ethical concerns. See the PLOS Data Policy and FAQ for detailed information.	Yes - all data are fully available without restriction

A Data Availability Statement describing where the data can be found is required at submission. Your answers to this question constitute the Data Availability Statement and **will be published in the article**, if accepted.<br><br>**Important:** Stating 'data available on request from the author' is not sufficient. If your data are only available upon request, select 'No' for the first question and explain your exceptional situation in the text box.<br><br>Do the authors confirm that all data underlying the findings described in their manuscript are fully available without restriction?	
**Describe where the data may be found in full sentences. If you are copying our sample text, replace any instances of XXX with the appropriate details.**<br><br>• If the data are **held or will be held in a public repository**, include URLs, accession numbers or DOIs. If this information will only be available after acceptance, indicate this by ticking the box below. For example: *All XXX files are available from the XXX database (accession number(s) XXX, XXX.*).<br>• If the data are all contained **within the manuscript and/or Supporting Information files**, enter the following: *All relevant data are within the manuscript and its Supporting Information files.*<br>• If neither of these applies but you are able to provide **details of access elsewhere**, with or without limitations, please do so. For example:<br><br>*Data cannot be shared publicly because of [XXX]. Data are available from the XXX Institutional Data Access / Ethics Committee (contact via XXX) for researchers who meet the criteria for access to confidential data.*<br><br>*The data underlying the results presented in the study are available from (include the name of the third party*	All rowdata files are available from the Seer database (https://seer.cancer.gov/registries/cancer_registry/index.html)

and contact information or URL).<br>• This text is appropriate if the data are owned by a third party and authors do not have permission to share the data.<br><br>* typeset	
Additional data availability information:	Tick here if your circumstances are not covered by the questions above and you need the journal's help to make your data available.

Powered by Editorial Manager® and ProduXion Manager® from Aries Systems Corporation

Manuscript Click here to access/download;Manuscript;Manuscript.docx

ORIGINAL RESEARCH

Delving into Female Breast Cancer: Distinct Disease-Specific Survival outcomes Between Invasive Lobular and Ductal Carcinomas revealed by Propensity Score Matching

Short title：Survival Differences Between Invasive Lobular and Ductal Carcinomas

Wu Zhang[1], Yuquan Huang[2], Ye Zhou[3], Jiaojiao Xue[4], Shan Gao[5], Lin Kang[6], Jian Shi[7], Tao Zhou[8], Yalong Duan[9], Sihan Guo[10],Qingxia Li[3*]

[1]Graduate School, North China University of Science and Technology, Tangshan, Hebei, China

[2]Guilin Medical University, Guilin, Guangxi, China

[3]The Fourth Department of Oncology, Hebei General Hospital, Shijiazhuang, China

[4]College of Postgraduate, Hebei Medical University, Shijiazhuang, China

[5]Department of Gland Surgery, Hebei General Hospital, Shijiazhuang, China

[6]Department of pathology, Hebei General Hospital, Shijiazhuang, China

[7]Department of Oncology, the Fourth Hospital of Hebei Medical University, Shijiazhuang, China

[8]Department of Breast Cancer Center, the Fourth Hospital of Hebei Medical University, Shijiazhuang, China

[9]School of Graduate Studies, Hebei North University, Zhangjiakou, China

[10] Department of Computer Science, Durham University, Durham, United Kingdom

* Corresponding author

Email: lqx73@163.com (QL)

Abstract

Purpose

The difference in prognosis between invasive lobular carcinoma (ILC) and invasive ductal carcinoma (IDC) is still controversial in the academic community. Resolving this controversy can help to more accurately determine patients' prognosis, provide further personalized treatment, alleviate unnecessary psychological burden for some patients, and provide direction for further fundamental research.

Patients and methods

A retrospective cohort study was conducted using the SEER Research Plus Data 8 Registries, Nov 2021 sub (1978-2019), including female breast cancer patients diagnosed with ILC or IDC between 2010 and 2015. Univariate and multivariate Cox regression analyses were performed, and key covariates affecting prognosis were selected. Propensity score matching (PSM) was employed to match patients, and balance tests were conducted to evaluate covariate distribution. Disease-specific survival (DSS) differences between the matched IDC and ILC groups were compared.

Results

Following PSM, the covariate differences between the IDC and ILC groups were significantly reduced. The survival analysis revealed a significantly better prognosis for the IDC group than the ILC group (Log-rank test $p < 0.001$), with a 28.0% increased risk observed in the ILC group.

Conclusion

This study provides evidence supporting the existence of significant differences in prognosis between IDC and ILC patients after rigorous matching. The IDC group displayed a significantly

better prognosis than the ILC group. Notably, these findings have implications for personalized treatment in clinical practice and contribute to the ongoing academic debate on survival differences between IDC and ILC. However, further research is needed to investigate the biological mechanisms, gene expression, and signaling pathway disparities between IDC and ILC, aiming to provide more targeted guidance for clinical decision-making.

Keywords: Invasive Ductal Carcinoma, Lobular Carcinomas, Prognosis, PSM

Introduction

According to GLOBOCAN 2020 estimates by the International Agency for Research on Cancer, female breast cancer has surpassed lung cancer as the most commonly diagnosed cancer (1). With the incidence of breast cancer increasing yearly, it has become one of the significant threats to women's health (2). Invasive lobular carcinoma (ILC) and invasive ductal carcinoma (IDC) are the two most common histological types of breast cancer, with ILC accounting for 10-15% of all breast cancers and IDC accounting for 70-80% of female breast tumors. Despite sharing similar etiologies, there are significant differences in biological characteristics, clinical manifestations, and prognosis between ILC and IDC (3-5). Thus, investigating the differences in Disease-Specific Survival (DSS) among different types of breast cancer patients holds great significance.

There is currently a debate in the academic community regarding the prognostic differences between ILC and IDC (6). In this study, we used propensity score matching (PSM) based on generalized linear models to analyze the differences in DSS rates between these two types of breast cancer patients. By comparing and balancing both patient groups' clinical characteristics, molecular subtypes, and treatment methods, we aim to reveal potential

prognostic differences and provide more accurate prognostic evaluations and treatment recommendations.

PSM is a widely used statistical method in observational studies to eliminate differences between two groups of patients with varying clinical and pathological characteristics, making the study results more reliable (7, 8). Additionally, the PSM method based on generalized linear models can adjust for patient background variables, achieving a balance between comparison groups and reducing bias caused by confounding factors (9, 10). Through an in-depth analysis of the prognostic differences between ILC and IDC, we aim to provide new evidence to resolve this controversy and bring new insights to clinical practice, ultimately improving breast cancer patients' survival and quality of life.

Material and methods

Study design and population

The data used in this study were obtained from the Surveillance, Epidemiology, and End Results (SEER) program, the primary program employed by the National Cancer Institute (NCI) to support cancer surveillance activities in the United States. The SEER program is an authoritative source of information on cancer incidence and survival (11). It collects and publishes cancer incidence and survival data from population-based cancer registries, covering approximately 48 percent of the U.S. population [https://seer.cancer.gov/registries/cancer_registry/index.html].

After obtaining research permission (Sequence Number: 24787-Nov2021), we utilized SEER*Stat version 8.4.0.1 to retrieve the SEER Research Plus Data 8 Registries. Nov 2021 sub (1978-2019) contains 4,765,822 cases, of which 4,351,209 are malignant cases. Since the

patient data provided by SEER is anonymized, no identifiable information is included in our analysis. Therefore, informed consent was not required.

All female patients included in our analysis had primary tumors in the breast and were diagnosed with positive histology, confirmed by diagnostic confirmation. The sources of the reports included hospital inpatient, outpatient or clinic, radiation therapy or oncology center (2006 and later), hospital or private laboratory, physician's office/private practice (LMD), nursing/convalescent home/hospice, and other hospital outpatient departments or surgical centers (2006 and later), excluding cases obtained from autopsies and those with only death certificate records. The cause of death (COD) and follow-up/survival months were available as complete dates, and samples with unknown causes of death and follow-up time were excluded. Furthermore, COD to site recode included alive and breast cancer-specific death patients. We also screened breast cancer patients with ICD-O-3 codes 8500/3: invasive ductal carcinoma and 8520/3: invasive lobular carcinoma.

Additionally, we obtained patients' age (<60 years, >=60 years), race (White, Black, Other - including Chinese, Japanese, Vietnamese, Korean, and other Asian ethnicities), marital status (Married, Divorced, Widowed, Single), primary site (Central, Upper Inner, Lower Inner, Upper Outer, Lower Outer, Overlapping, Others), grade (I, II, III, IV), summary stage (Localized, Regional, Distant), AJCC stage (I, II, III, IV), laterality (Right, Left), surgical and radiation sequence (including PORT, NROS, others), systemic surgery sequence (including AAT, NSOST, others), ER/PR status (positive, negative), tumor size (<=1cm, <=2cm, <=3cm, <=4cm, <=5cm, >5cm), and subtype (HR+/HER2+, HR+/HER2-, HR-/HER2+, HR-/HER2-) from the SEER database. Overall, a total of 66,405 patient samples were collected for our analysis.

Statistical analysis

We divided the dataset into two groups based on histology: the IDC group and the ILC group. To ensure the accuracy and reliability of the analysis, we first used the ks.test function in R version 4.2.1 to perform a Kolmogorov-Smirnov (KS) test on the only continuous variable-survival to select an appropriate inter-group comparison analysis method. Although the Shapiro-Wilk test is considered a better normality test method, it has higher sensitivity to small sample sizes. When the sample size is large, the Shapiro-Wilk test may result in a misjudgment of P-values (12). Thus, the KS test is more suitable (13). Based on the results of the KS test (D = 0.053628, p-value < 0.001), we compared the between-group differences in continuous non-normally distributed variables using the Mann-Whitney U test. Then, we described the sample distribution characteristics of this type of variable using the median (interquartile range) (14). We used the chi-square test for other categorical variables, which is suitable for large sample sizes, to compare the distribution differences between the IDC and ILC groups. Moreover, we calculated the corresponding P-values and described the distribution of samples within each group using frequency (percentage) (15). Through this method, we could fully understand the differences in various variables between the IDC and ILC groups, providing a strong basis for the subsequent analysis using the PSM method based on generalized linear models.

Before PSM, we considered the presence of confounding factors. To reduce bias risks and achieve better-matching results, we identified important variables affecting prognosis through Cox regression analysis. We included them as input variables in the PSM model. Specifically, we used the coxph function of the survival package to perform univariate Cox regression analysis for the IDC and ILC groups, respectively, and included covariates with significant

statistical differences ($P < 0.050$) in subsequent multivariate Cox regression analysis. Based on the potential interaction between covariates, we further estimated the independent prognostic ability of each variable. We then evaluated the relationship between each variable and survival by calculating the hazard ratio, 95% confidence interval, and P-value for both univariate and multivariate variable analyses. Furthermore, we excluded variables with P-values greater than 0.050 in univariate and multivariate Cox regression analysis results for the IDC and ILC groups. We also used the remaining variables as covariates closely related to survival for subsequent PSM to minimize the weakening of validity and unnecessary sample loss brought about by including too many irrelevant variables.

Afterward, we constructed a binary logistic regression model with histology as the dependent variable and the covariates identified from the Cox regression analysis as independent variables using the generalized linear model (GLM) function of the matchit package. The propensity score value, representing the probability of each individual being assigned to the IDC or ILC group, was calculated for each individual. Then, using the nearest neighbor matching algorithm with a caliper matching threshold of 0.0001 and a 1:1 ratio, we matched IDC and ILC individuals based on their propensity scores and randomized the matching order to improve matching precision and reduce confounding bias. Thus, this improves the quality of comparing the two pathological types regarding the difference in prognosis and risk. To further screen the variables that had a more significant impact on the grouping, we subsequently used the love.plot function of the cobalt package to conduct a balance test after matching, setting a threshold of 0.05. We repeated the comparison of differences between different variables in the IDC and ILC groups. After matching, we plotted

the baseline table of clinical data to verify whether the differences between the groups were effectively controlled and ensure the reliability of the study results. Finally, we compared the prognosis differences between the matched IDC and ILC groups using the survival function of the R package survival. Furthermore, we validated the significance of the differences in prognosis between the two groups using three statistical methods: the log-rank test, the Wald test, and the likelihood ratio test.

Results

We obtained 510,385 primary breast cancer samples from the SEER Research Plus Data 8 Registries. Nov 2021 sub (1978-2019), of which 66,405 female breast cancer patients were included in our analysis after excluding and including samples according to our criteria. Of these, 58,497 cases were IDC (88.1%), and 7,908 were ILC (11.9%). Additionally, there were 5,726 cases of breast cancer-specific deaths (9.9%) in the IDC group and 853 cases (10.8%) in the ILC group. The median survival time in the IDC group was 75.0 (58.0, 95.0) months, while in the ILC group, it was 73.0 (57.0, 93.0) months. Moreover, the distributions of age, race, marital status, primary tumor site, molecular subtype, pathological grade, summary stage, AJCC, order of surgery and systemic therapy, order of surgery and radiation therapy, hormone status, and tumor size were statistically significantly different between the IDC and ILC groups. Notably, the proportion of elderly patients in the ILC group (61.1%) was significantly higher than that in the IDC group (50.8%). Regarding molecular subtypes, the HR+/HER2- subtype accounted for 94.0% of the ILC group. In contrast, it was only 72.0% in the IDC group, and the other three subtypes were lower in proportion in the ILC group than in the IDC group. In terms of pathological grade, the proportion of Grade I (31.6%) and Grade II (61.3%) in the ILC group

was significantly higher than that in the IDC group (23.4% and 41.5%, respectively). In comparison, the proportion of Grade III (35.0%) and Grade IV (0.1%) in the IDC group was significantly higher than that in the ILC group (Grade III: 7.1%, Grade IV: 0.0%). Furthermore, the proportions of ER-positive (98.1%) and PR-positive (83.5%) patients in the ILC group were significantly higher than those in the IDC group (ER: 82.2%, PR: 71.8%) in terms of hormone status. For tumor size, the proportion of tumors with a diameter of 2cm or less was higher in the IDC group than in the ILC group (64.8% vs. 51.0%). In comparison, the proportion of tumors with a diameter of 2cm or more in the ILC group was higher than that in the IDC group. (Table 1).

Table 1. Clinical data Baseline table before propensity score matching

level	Overall	IDC	ILC	p
n	66405	58497	7908	
survival (median [IQR])	75.0 [57.0, 95.0]	75.0 [58.0, 95.0]	73.0 [57.0, 93.0]	<0.001
status (%)				
Alive	59826 (90.1)	52771 (90.2)	7055 (89.2)	0.006
Dead	6579 (9.9)	5726 (9.8)	853 (10.8)	
age (%)				
<60	31885 (48.0)	28806 (49.2)	3079 (38.9)	<0.001
>=60	34520 (52.0)	29691 (50.8)	4829 (61.1)	
race (%)				
White	55083 (83.0)	48103 (82.2)	6980 (88.3)	<0.001
Black	5879 (8.9)	5339 (9.1)	540 (6.8)	
Other	5443 (8.2)	5055 (8.6)	388 (4.9)	
Maritial status (%)				
Married	40847 (61.5)	35952 (61.5)	4895 (61.9)	<0.001
Divorced	7807 (11.8)	6880 (11.8)	927 (11.7)	

Widowed	7308 (11.0)	6299 (10.8)	1009 (12.8)	
single	10443 (15.7)	9366 (16.0)	1077 (13.6)	
Primary Site (%)				
Central	2755 (4.2)	2376 (4.1)	379 (4.8)	<0.001
Upper inner	8704 (13.1)	7869 (13.5)	835 (10.6)	
Lower inner	3787 (5.7)	3466 (5.9)	321 (4.1)	
Upper outer	23034 (34.7)	20256 (34.6)	2778 (35.1)	
Lower outer	5270 (7.9)	4659 (8.0)	611 (7.7)	
Overlapping	15453 (23.3)	13621 (23.3)	1832 (23.2)	
others	7402 (11.2)	6250 (10.7)	1152 (14.6)	
Subtype (%)				
HR+/HER2+	7020 (10.6)	6687 (11.4)	333 (4.2)	<0.001
HR+/HER2-	49513 (74.6)	42081 (72.0)	7432 (94.0)	
HR-/HER2+	2844 (4.3)	2807 (4.8)	37 (0.5)	
HR-/HER2-	7028 (10.6)	6922 (11.8)	106 (1.3)	
Grade (%)				
I	16173 (24.4)	13678 (23.4)	2495 (31.6)	<0.001
II	29097 (43.8)	24247 (41.5)	4850 (61.3)	
III	21054 (31.7)	20494 (35.0)	560 (7.1)	
IV	81 (0.1)	78 (0.1)	3 (0.0)	
Summary stage (%)				
Localized	44707 (67.3)	39655 (67.8)	5052 (63.9)	<0.001
Regional	19241 (29.0)	16713 (28.6)	2528 (32.0)	
Distant	2457 (3.7)	2129 (3.6)	328 (4.2)	
AJCC (%)				
I	36096 (54.4)	32596 (55.7)	3500 (44.3)	<0.001
II	21498 (32.4)	18585 (31.8)	2913 (36.8)	
III	6458 (9.7)	5280 (9.0)	1178 (14.9)	
IV	2353 (3.5)	2036 (3.5)	317 (4.0)	
Laterality (%)				

Right	32886 (49.5)	29037 (49.6)	3849 (48.7)	0.109
Left	33519 (50.5)	29460 (50.4)	4059 (51.3)	
Systemic Sur Seq (%)				
AAT	45514 (68.5)	39718 (67.9)	5796 (73.3)	<0.001
NSOST	13284 (20.0)	11795 (20.2)	1489 (18.8)	
others	7607 (11.5)	6984 (11.9)	623 (7.9)	
Surg Rad Seq (%)				
PORT	37712 (56.8)	33461 (57.2)	4251 (53.8)	<0.001
NROS	27921 (42.1)	24319 (41.6)	3602 (45.6)	
others	772 (1.2)	717 (1.2)	55 (0.7)	
ER (%)				
Positive	55861 (84.1)	48107 (82.2)	7754 (98.1)	<0.001
Negtive	10544 (15.9)	10390 (17.8)	154 (2.0)	
PR (%)				
Positive	48625 (73.2)	42019 (71.8)	6606 (83.5)	<0.001
Negtive	17780 (26.8)	16478 (28.2)	1302 (16.5)	
Tumor size (%)				
<=1	18397 (27.7)	16822 (28.8)	1575 (20.0)	<0.001
<=2	23557 (35.5)	21096 (36.1)	2461 (31.1)	
<=3	12165 (18.3)	10656 (18.2)	1509 (19.1)	
<=4	5000 (7.5)	4320 (7.4)	680 (8.6)	
<=5	2559 (3.9)	2092 (3.6)	467 (5.9)	
> 5	4727 (7.1)	3511 (6.0)	1216 (15.4)	

Abbreviation: AAT, Adjuvant Therapy; NSOST, No systemic therapy and/or surgical therapy; PORT, Post-Operative Radiation Therapy; NROS, No radiation and/or cancer-directed surgery.

The univariate and multivariate Cox regression analysis results of IDC and ILC patients are summarized in S1 and S2 Tables. From the univariate Cox regression analysis of IDC,

laterality showed no significant independent prognostic ability (P=0.747), and the independent prognostic efficacy of the other subcategories in the surgery and radiation therapy sequence was insignificant (P=0.143). From the multivariate Cox regression analysis, none of the seven Primary_site prognostic abilities were significant, and the prognostic ability of the HR-/HER2- subtype was insignificant (P=0.743). The prognostic efficacy of the other subcategories in the surgery and radiation therapy sequence was also insignificant (P=0.260) (S1 Table).

From the univariate Cox regression analysis of ILC, the independent prognostic efficacy of the other subcategories in Primary_site was insignificant (P=0.263). Laterality showed no significant independent prognostic ability (P=0.633), and the independent prognostic efficacy of the HR+/HER2- subtype was insignificant (P=0.118). From the multivariate Cox regression analysis of ILC, Black patients and others showed no significant prognostic ability compared to White patients. The single marital status showed no significant prognostic efficacy (P=0.490). Additionally, none of the seven different Primary site prognostic abilities were significant, consistent with the multiple regression analysis of IDC. The prognostic efficacy of the other subcategories in the surgery and radiation therapy sequence in ILC was insignificant (P=0.780), and the prognostic efficacy of the HR+/HER2- subtype was insignificant (P=0.199). Furthermore, tumor size between 2 and 3 cm and greater than 5 cm did not show a clear prognostic association compared to a tumor size of 1 cm (S2 Table).

The prognostic ability of Grade IV was insignificant in both univariate and multivariate Cox regression analysis. Still, we consider this to be due to the small number of Grade IV samples in ILC rather than its true situation. Finally, we included variables other than laterality

that had no significant prognostic ability in both univariate and multivariate Cox regression results in the calculation of the propensity score.

We first calculated the absolute standardized mean difference (AMD) of each covariate from the balance test of covariates before and after PSM. Notably, AMD is a measure for evaluating the degree of distributional differences of covariates before and after matching (16), and the absolute mean difference graph of covariates was plotted (Fig 1, left panel). It can be seen from the graph that the absolute standardized mean differences between each covariate of IDC and ILC groups were all less than 0.050 after PSM, suggesting that differences between each covariate in the two groups were significantly reduced after matching. Moreover, in the baseline data distribution after PSM (Table 2), there were no significant differences observed in the distribution of all variables related to DSS between IDC and ILC groups (P>0.050). However, the absolute standardized mean differences of covariates such as age, AJCC stage, grade classification, ER and PR status, molecular subtypes, and tumor size were all greater than 0.05 before PSM, indicating significant differences in these covariates' distribution.

Table 2. Baseline clinical data after propensity score matching.

Characteristic	Overall	IDC	ILC	p
NO.	12794	6397	6397	
survival (median [IQR])	75.000 [58.0, 95.0]	76.000 [59.0, 96.0]	75.000 [58.0, 94.0]	0.005
status (%)				
Alive	11821 (92.4)	5944 (92.9)	5877 (91.9)	0.028
Dead	973 (7.6)	453 (7.1)	520 (8.1)	
age (%)				
<60	5216 (40.8)	2625 (41.0)	2591 (40.5)	0.553
>=60	7578 (59.2)	3772 (59.0)	3806 (59.5)	

race (%)				
Black	690 (5.4)	344 (5.4)	346 (5.4)	0.976
Other	633 (5.0)	314 (4.9)	319 (5.0)	
White	11471 (89.6)	5739 (89.7)	5732 (89.6)	
Maritial status (%)				
Divorced	1385 (10.8)	699 (10.9)	686 (10.7)	0.979
Married	8283 (64.7)	4140 (64.7)	4143 (64.8)	
single	1654 (12.9)	827 (12.9)	827 (12.9)	
Widowed	1472 (11.5)	731 (11.4)	741 (11.6)	
Primary Site (%)				
Central	516 (4.0)	261 (4.1)	255 (4.0)	0.999
Lower outer	962 (7.5)	481 (7.5)	481 (7.5)	
Lower inner	508 (4.0)	256 (4.00)	252 (3.9)	
others	1675 (13.1)	826 (12.9)	849 (13.3)	
Overlapping	2996 (23.4)	1502 (23.5)	1494 (23.4)	
Upper outer	4733 (37.0)	2368 (37.0)	2365 (37.0)	
Upper inner	1404 (11.0)	703 (11.0)	701 (11.0)	
Subtype (%)				
HR-/HER2-	181 (1.4)	85 (1.3)	96 (1.5)	0.711
HR-/HER2+	66 (0.5)	34 (0.5)	32 (0.5)	
HR+/HER2-	11984 (93.7)	5987 (93.6)	5997 (93.8)	
HR+/HER2+	563 (4.4)	291 (4.6)	272 (4.3)	
Grade (%)				
I	3725 (29.1)	1833 (28.7)	1892 (29.6)	0.412
II	7977 (62.4)	4004 (62.6)	3973 (62.1)	
III	1092 (8.5)	560 (8.8)	532 (8.3)	
AJCC (%)				
I	6833 (53.4)	3427 (53.6)	3406 (53.2)	0.400
II	4354 (34.0)	2186 (34.2)	2168 (33.9)	
III	1279 (10.0)	635 (9.9)	644 (10.1)	

IV	328 (2. 6)	149 (2.3)	179 (2.8)	
Laterality (%)				
Left	6372 (49.8)	3066 (47.9)	3306 (51.7)	<0.001
Right	6422 (50.2)	3331 (52.1)	3091 (48.3)	
Systemic Sur Seq (%)				
AAT	9643 (75.4)	4846 (75.8)	4797 (75.0)	0.588
NSOST	2402 (18.8)	1185 (18.5)	1217 (19.0)	
others	749 (5.9)	366 (5.7)	383 (6.0)	
Surg Rad Seq (%)				
NROS	5732 (44.8)	2860 (44.7)	2872 (44.9)	0.541
others	71 (0.6)	31 (0.5)	40 (0.6)	
PORT	6991 (54.6)	3506 (54.8)	3485 (54.5)	
ER (%)				
Negtive	266 (2.1)	127 (2.0)	139 (2.2)	0.496
Positive	12528 (97.9)	6270 (98.0)	6258 (97.8)	
PR (%)				
Negtive	1669 (13.6)	836 (13.1)	833 (13.0)	0.958
Positive	11125 (87.0)	5561 (86.9)	5564 (87.0)	
Tumor size (%)				
<=1	3041 (23.8)	1514 (23.7)	1527 (23.9)	0.937
<=2	4655 (36.4)	2317 (36.2)	2338 (36.6)	
<=3	2622 (20.5)	1323 (20.7)	1299 (20.3)	
<=4	944 (7.4)	464 (7.3)	480 (7.5)	
<=5	470 (3.7)	243 (3.8)	227 (3.6)	
> 5	1062 (8.3)	536 (8.4)	526 (8.2)	

Abbreviation: AAT, Adjuvant Therapy; NSOST, No systemic therapy and/or surgical therapy; PORT, Post-Operative Radiation Therapy; NROS, No radiation and/or cancer-directed surgery.

Next, we analyzed the differences between each covariate in the cumulative distribution functions and applied the KS statistic. The KS statistic is a non-parametric test method to measure the difference between two distributions. It is calculated as the maximum distance between the cumulative distribution functions of the two distributions (17). In this study, we calculated the KS statistic for each covariate before and after matching (Fig 1, right panel). The KS statistic of covariates such as age, AJCC stage, grade classification, ER and PR status, molecular subtypes, and tumor size were all greater than 0.05 before PSM, indicating that the distribution of these covariates within the sample before matching was unbalanced. However, after PSM, the KS statistic of each covariate was less than 0.05, almost approaching 0, consistent with the distribution of absolute mean difference, implying that the sample was balanced on these covariates after matching.

Fig 1. Covariate Balance Test Plot after Propensity Score Matching.

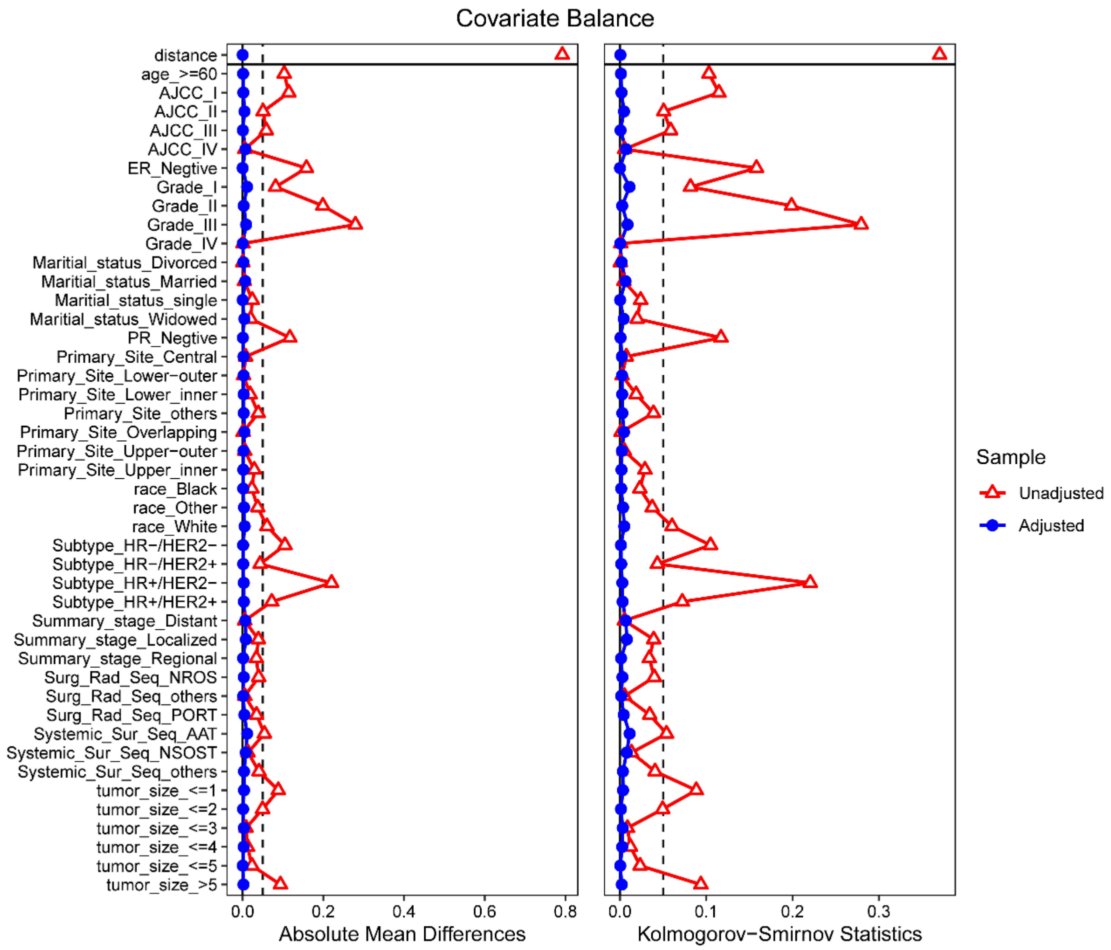

Finally, we conducted a survival analysis on the matched IDC and ILC groups. The KM survival analysis results showed that the prognosis of the IDC group was significantly better than that of the ILC group (Log-rank test p < 0.001) (Fig 2). Compared with the IDC group, Cox proportional hazards regression analysis showed that the risk of the ILC group was 28.0% higher than the IDC group (HR=1.28, CI=1.12-1.45). Meanwhile, the results of the likelihood ratio test (W value: 1.96, p < 0.001) and Wald test (W value: 11.87, p= < 0.001) were consistent with the Log-rank test results, further confirming the significant difference in prognosis between the IDC and ILC groups.

Fig 2. KM Survival analysis of IDC and ILC groups after PSM.

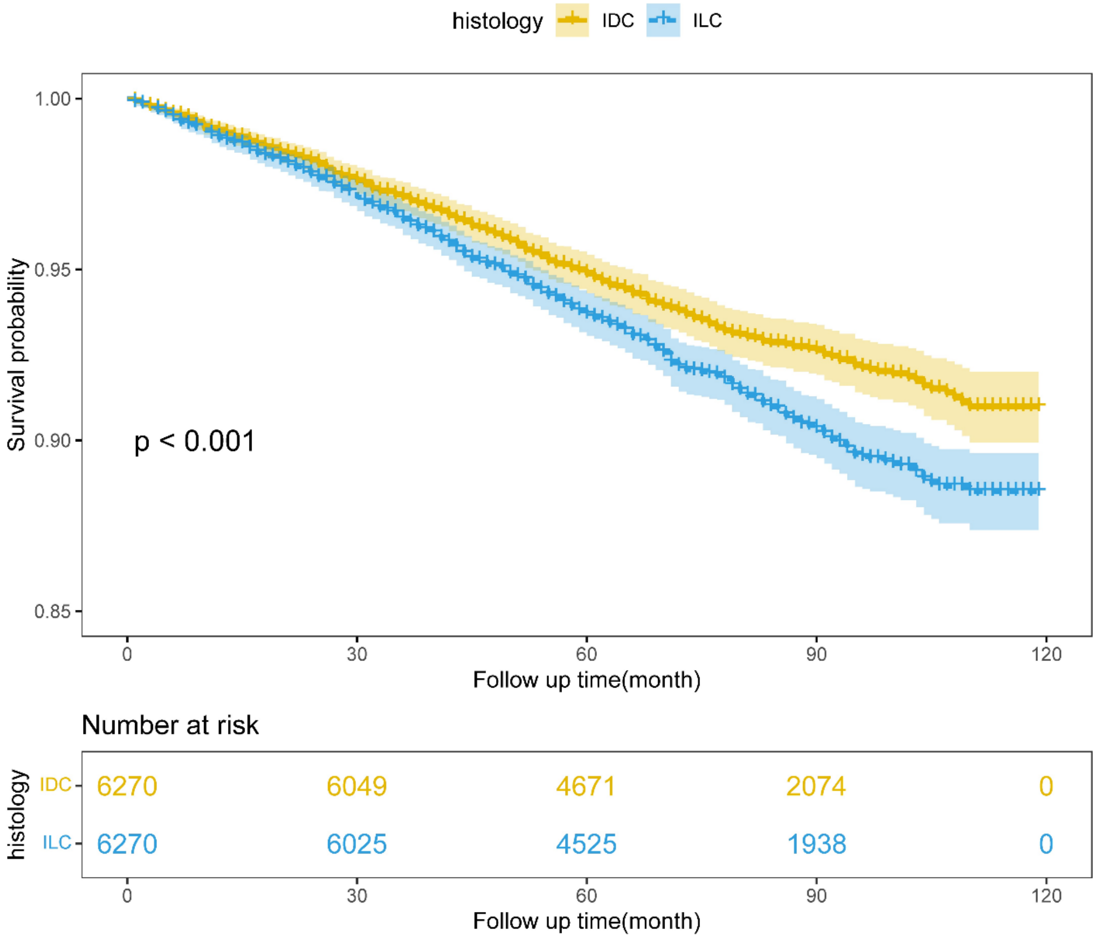

Discussion

IDC is the most prevalent breast cancer type, believed to primarily originate from malignant epithelial cells of the breast's terminal ducts(18). IDC is characterized by invasive growth, with tumor cells infiltrating the breast stroma and spreading after breaking through ductal walls. Microscopically, tumor cells often form cord-like, cluster-like, or small beam-like patterns. Clinically, IDC manifests mainly as a nipple-like and solid mass. IILC, the second most common invasive breast cancer histological type after IDC, originates primarily from malignant cells beneath the breast lobule's epithelium (19). Microscopically, ILC often exhibits small, uniform, and non-adhesive cancer cells distributed individually or diffusely in the fibrous

stroma (20). Compared to IDC, ILC lacks apparent clinical masses and has a lower detection rate on breast ultrasound and X-ray examinations (21).

Besides the aforementioned histological differences, ILC and IDC also display molecular disparities. ILC patients lack E-cadherin expression, a deficiency that stimulates the activation signal of Epithelial-Mesenchymal Transition (EMT), resulting in a higher incidence of bone, gastrointestinal, omental, and ovarian metastases compared to IDC patients (22-25). Moreover, studies have revealed that genomic profiles of ILC and IDC patients differ based on tissue sequencing assessments. ILC patients exhibit a higher tumor mutation burden and variations in immune infiltration and copy number changes(26-29). Furthermore, even though the clinical treatment methods for both are relatively similar, some differences still exist. For instance, in clinical practice, the frequency of ILC patients undergoing mastectomy is slightly higher than that of IDC patients (24). Furthermore, there is ongoing debate regarding the clinical outcomes of ILC and IDC patients. Wasif et al. discovered that ILC patients had a significantly higher five-year DSS rate compared to IDC patients ($P < 0.001$) through stage-matched analysis(30). Chen et al. obtained similar results, with ILC patients having a survival advantage within five years post-surgery compared to IDC patients but demonstrating a worse long-term prognosis [24]. Furthermore, Yang et al. found no significant difference in overall survival rates between ILC and IDC patients during the first five years after PMS analysis [25]. In contrast, some studies have reported that ILC patients have a higher ten-year overall survival rate than IDC patients(31, 32).

Considering the abovementioned research, we hypothesize that the differences in study outcomes may stem from variations in the chosen study populations, variable control, and

distinct data analysis methods. Therefore, to provide more comprehensive and precise data, this study matched with a more comprehensive set of covariates and their subclasses, eliminating the impact of baseline patient characteristics. Using PSM based on a generalized linear model, we explored the disparities in DSS between these two types of breast cancer patients. Through balancing tests of covariates before and after matching, the disparities in various covariates between the IDC and ILC groups were significantly reduced, offering a more balanced foundation for subsequent survival analysis. From the survival analysis post-PSM, we discovered that the prognosis of the IDC group was significantly better than that of the ILC group (Log-rank test $p < 0.001$), and the risk of the ILC group increased by 28.0% compared to the IDC group. This result indicates that after PSM, the difference in tumor-specific survival between IDC and ILC patients is substantial, supporting the notion that IDC and ILC have distinct clinical outcomes. In contrast, a recent study conducted by Ciqiu Yang et al. showed that prior to PSM, the overall survival (OS) of IDC patients was superior to that of ILC patients (HR=1.045, P=0.025, 95%CI: 1.007-1.085). However, after PSM, the difference in OS between the two groups disappeared. In addition to the reasons for these divergent results mentioned above, the differing focus outcomes may be one of the leading causes of such discrepancies. For breast cancer patients with longer survival periods, DSS can help us avoid the influences of other potential interfering factors more effectively than OS, thereby reflecting the impact of breast cancer on survival more accurately.

This study has some noted limitations. As a retrospective study, the data is sourced from the SEER database. Although this is an authoritative large-scale cancer registry database, its population coverage primarily comprises white individuals in the United States. To further

verify the universality and representativeness of these conclusions, it is necessary to expand the sample sources and scope for patients from other regions and ethnicities. Additionally, although we have substantially reduced biases through PSM, there may still be unobserved potential confounding factors that could affect the accuracy of the results.

Conclusion

Through applying PSM, our study discovered that, upon stringent matching, the prognosis for patients in the IDC group was markedly superior to those in the ILC group. This finding offers compelling evidence to support individualized treatment strategies for patients with IDC and ILC in clinical settings. Nevertheless, it remains essential for future research to delve deeper into the distinctions between IDC and ILC concerning biological mechanisms, gene expression patterns, and signaling pathways to provide more precise and targeted guidance in clinical practice.

Acknowledgments

We thank all medical students who devoted their time to this study. The authors thank AiMi Academic Services (www.aimieditor.com) for English language editing and review services.

Author contributions

Wu Zhang, Data Curation, Formal Analysis, Software, Investigation, Writing-Original Draft Preparation, Writing-Review & Editing, Yuquan Huang, Formal Analysis, Software, Visualization, Writing-Original Draft Preparation, Ye Zhou, Supervision, Visualization, Writing-Original Draft Preparation, Jiaojiao Xue, Validation, Writing-Original Draft Preparation, Shan Gao, Supervision, Writing-Original Draft Preparation, Lin Kang, upervision,

Validation, Jian Shi, Supervision, Writing-Review & Editing, Tao Zhou, Project Administration, Supervision, Yalong Duan, Formal Analysis, Visualization, Sihan Guo, Validation, Formal Analysis, Writing-Review & Editing, Qingxia Li, Funding Acquisition, Project Administration, Writing-Original Draft Preparation, Writing-Review & Editing

References

1. Sung H, Ferlay J, Siegel RL, Laversanne M, Soerjomataram I, Jemal A, et al. Global Cancer Statistics 2020: GLOBOCAN Estimates of Incidence and Mortality Worldwide for 36 Cancers in 185 Countries. CA Cancer J Clin. 2021;71(3):209-49.
2. Kashyap D, Pal D, Sharma R, Garg VK, Goel N, Koundal D, et al. Global Increase in Breast Cancer Incidence: Risk Factors and Preventive Measures. Biomed Res Int. 2022;2022:9605439.
3. O'Connor DJ, Davey MG, Barkley LR, Kerin MJ. Differences in sensitivity to neoadjuvant chemotherapy among invasive lobular and ductal carcinoma of the breast and implications on surgery-A systematic review and meta-analysis. Breast. 2022;61:1-10.
4. Thornton MJ, Williamson HV, Westbrook KE, Greenup RA, Plichta JK, Rosenberger LH, et al. Neoadjuvant Endocrine Therapy Versus Neoadjuvant Chemotherapy in Node-Positive Invasive Lobular Carcinoma. Ann Surg Oncol. 2019;26(10):3166-77.
5. Mueller C, Haymond A, Davis JB, Williams A, Espina V. Protein biomarkers for subtyping breast cancer and implications for future research. Expert Rev Proteomics. 2018;15(2):131-52.
6. Dalenc F, Lusque A, De La Motte Rouge T, Pistilli B, Brain E, Pasquier D, et al. Impact of lobular versus ductal histology on overall survival in metastatic breast cancer: a French

retrospective multicentre cohort study. Eur J Cancer. 2022;164:70-9.

7. Zhang Z, Kim HJ, Lonjon G, Zhu Y, written on behalf of AMEB-DCTCG. Balance diagnostics after propensity score matching. Ann Transl Med. 2019;7(1):16.

8. Wang J. To use or not to use propensity score matching? Pharm Stat. 2021;20(1):15-24.

9. Bi G, Liang J, Shan G, Zhan C. Propensity Score Matching for Bias Reduction in Genomic Profiling. J Clin Oncol. 2022;40(11):1259-60.

10. Morgan CJ. Reducing bias using propensity score matching. J Nucl Cardiol. 2018;25(2):404-6.

11. Hossain MJ, Xie L, Caywood EH. Prognostic factors of childhood and adolescent acute myeloid leukemia (AML) survival: evidence from four decades of US population data. Cancer Epidemiol. 2015;39(5):720-6.

12. Mishra P, Pandey CM, Singh U, Gupta A, Sahu C, Keshri A. Descriptive statistics and normality tests for statistical data. Ann Card Anaesth. 2019;22(1):67-72.

13. Damiani G, Bragazzi NL, Garbarino S, Chattu VK, Shapiro CM, Pacifico A, et al. Psoriatic and psoriatic arthritis patients with and without jet-lag: does it matter for disease severity scores? Insights and implications from a pilot, prospective study. Chronobiol Int. 2019;36(12):1733-40.

14. Kim GS, Smith AK, Xue F, Michopoulos V, Lori A, Armstrong DL, et al. Methylomic profiles reveal sex-specific differences in leukocyte composition associated with post-traumatic stress disorder. Brain Behav Immun. 2019;81:280-91.

15. McCloskey RJ, Pei F. The role of parenting stress in mediating the relationship between neighborhood social cohesion and depression and anxiety among mothers of young children in fragile families. J Community Psychol. 2019;47(4):869-81.

16. Austin PC. The relative ability of different propensity score methods to balance measured covariates between treated and untreated subjects in observational studies. Med Decis Making. 2009;29(6):661-77.

17. Aslam M. Introducing Kolmogorov-Smirnov Tests under Uncertainty: An Application to Radioactive Data. ACS Omega. 2020;5(1):914-7.

18. Chen X, Li X, Wang J, Zhao L, Peng X, Zhang C, et al. Breast invasive ductal carcinoma diagnosis with a three-miRNA panel in serum. Biomark Med. 2021;15(12):951-963.

19. Danzinger S, Hielscher N, Izso M, Metzler J, Trinkl C, Pfeifer C, et al. Invasive lobular carcinoma: clinicopathological features and subtypes. J Int Med Res. 2021;49(6):3000605211017039.

20. Tan PH, Ellis I, Allison K, Brogi E, Fox SB, Lakhani S, et al. The 2019 World Health Organization classification of tumours of the breast. Histopathology. 2020;77(2):181-5.

21. Veltman J, Boetes C, van Die L, Bult P, Blickman JG, Barentsz JO. Mammographic detection and staging of invasive lobular carcinoma. Clin Imaging. 2006;30(2):94-8.

22. Siegel RL, Miller KD, Fuchs HE, Jemal A. Cancer Statistics, 2021. CA Cancer J Clin. 2021;71(1):7-33.

23. Rakha EA, Ellis IO. Lobular breast carcinoma and its variants. Semin Diagn Pathol. 2010;27(1):49-61.

24. Arpino G, Bardou VJ, Clark GM, Elledge RM. Infiltrating lobular carcinoma of the breast: tumor characteristics and clinical outcome. Breast Cancer Res. 2004;6(3):R149-56.

25. Norgard RJ, Pitarresi JR, Maddipati R, Aiello-Couzo NM, Balli D, Li J, et al. Calcium signaling induces a partial EMT. EMBO Rep. 2021;22(9):e51872.

26. Sokol ES, Feng YX, Jin DX, Basudan A, Lee AV, Atkinson JM, et al. Loss of function of NF1 is a mechanism of acquired resistance to endocrine therapy in lobular breast cancer. Ann Oncol. 2019;30(1):115-23.

27. Desmedt C, Salgado R, Fornili M, Pruneri G, Van den Eynden G, Zoppoli G, et al. Immune Infiltration in Invasive Lobular Breast Cancer. J Natl Cancer Inst. 2018;110(7):768-76.

28. Loo LW, Grove DI, Williams EM, Neal CL, Cousens LA, Schubert EL, et al. Array comparative genomic hybridization analysis of genomic alterations in breast cancer subtypes. Cancer Res. 2004;64(23):8541-9.

29. Davis AA, Gerratana L, Clifton K, Medford AJ, Velimirovic M, Hensing WL, et al. Circulating tumour DNA characterisation of invasive lobular carcinoma in patients with metastatic breast cancer. EBioMedicine. 2022;86:104316.

30. Wasif N, Maggard MA, Ko CY, Giuliano AE. Invasive lobular vs. ductal breast cancer: a stage-matched comparison of outcomes. Ann Surg Oncol. 2010;17(7):1862-9.

31. Adachi Y, Ishiguro J, Kotani H, Hisada T, Ichikawa M, Gondo N, et al. Comparison of clinical outcomes between luminal invasive ductal carcinoma and luminal invasive lobular carcinoma. BMC Cancer. 2016;16:248.

32. P R. Rosen's breast pathology. 3. Philadelphia: Lippincott williams & wilkins. 2009.

Supporting information

S1 Table. IDC group univariate and multivariate Cox regression analysis.

S2 Table. ILC group univariate and multivariate Cox regressionanalysis.

Fig1.tif

Click here to access/download;Figure;Fig1.tif

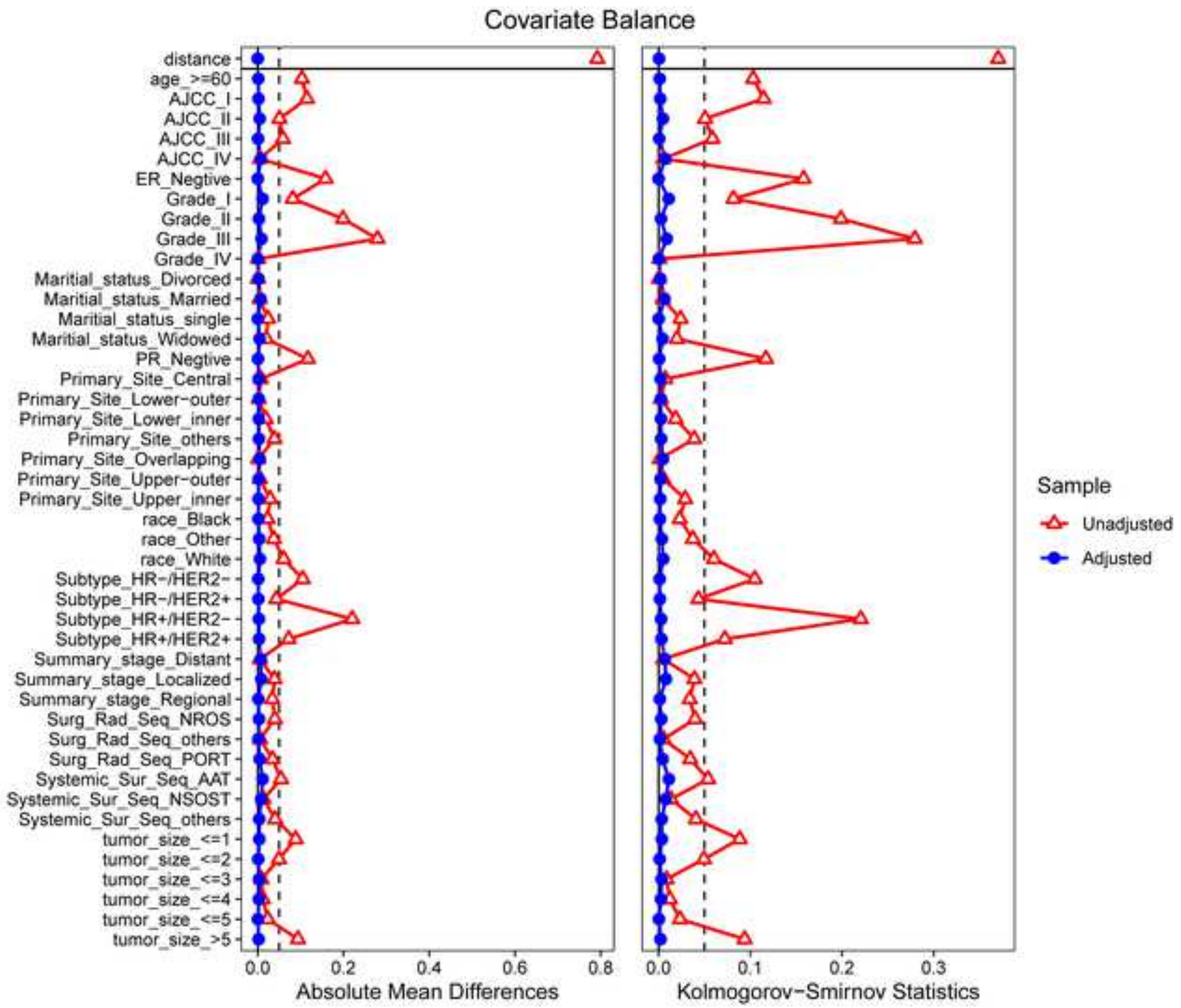

Click here to access/download;Figure;Fig2.tif

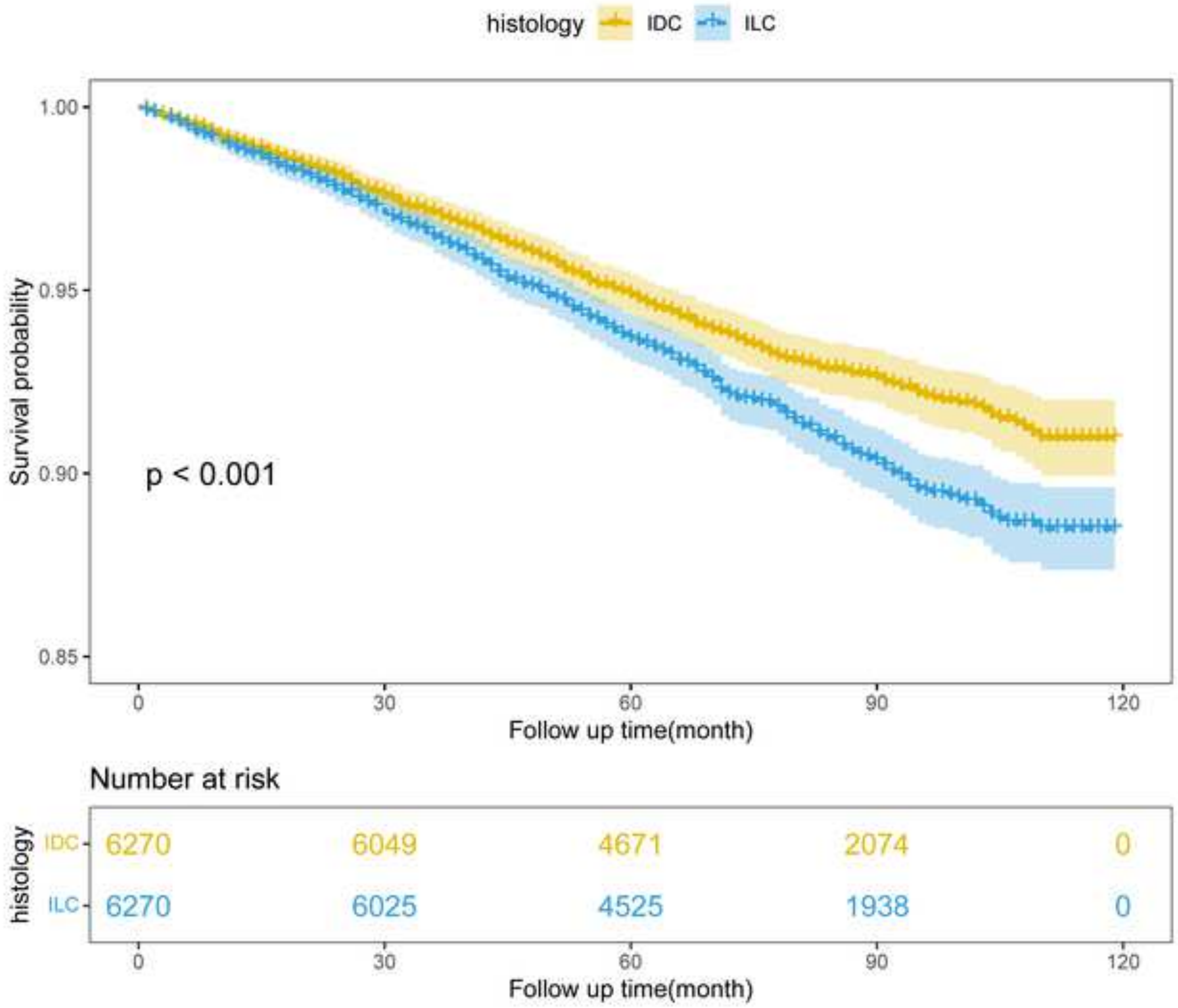

Click here to access/download
Supporting Information
Supporting information.docx

ORIGINAL RESEARCH

~~Wu Zhang et al~~

Delving into ~~Female~~Female Breast Cancer: ~~A New Perspective on~~Distinct Disease-Specific Survival ~~Differences~~outcomes Between Invasive Lobular and Ductal Carcinomas ~~Revealed~~revealed by Propensity Score Matching

Short title: Survival Differences Between Invasive Lobular and Ductal Carcinomas

Wu Zhang[1], Yuquan Huang[2], Ye Zhou[3], Jiaojiao Xue[4], Shan Gao[5], Lin Kang[6], Jian Shi[7], Tao Zhou[8], Yalong Duan[9], Sihan Guo[10] Qingxia Li[3,*]

~~[1] Graduate~~

[1]Graduate School, North China University of Science and Technology, Tangshan, Hebei, China

~~[2] Guilin~~[2]Guilin Medical University, Guilin, Guangxi, China

~~[3] The~~[3]The Fourth Department of Oncology, Hebei General Hospital, Shijiazhuang, China

~~[4] College~~[4]College of Postgraduate, Hebei Medical University, Shijiazhuang, China

[5]Department of Gland Surgery, Hebei General Hospital, Shijiazhuang, China

[6]Department of pathology, Hebei General Hospital, Shijiazhuang, China

[7]Department of Oncology, the Fourth Hospital of Hebei Medical University, Shijiazhuang, China

[8]Department of Breast Cancer Center, the Fourth Hospital of Hebei Medical University, Shijiazhuang, China

[9]School of Graduate Studies, Hebei North University, Zhangjiakou, China

~~Correspondence: Qingxia Li~~

~~Dept. of Oncology,Hebei General Hospital,348 Heping West Road,Shijiazhuang 050051,~~

Formatted: Font: Times New Roman, 11 pt
Formatted: Line spacing: Double
Formatted: Font: Times New Roman, 18 pt, Bold
Formatted: Normal, Line spacing: Double
Formatted: Font: Times New Roman, 18 pt, Bold
Formatted: Font: Times New Roman, 18 pt, Bold
Formatted: Font: Times New Roman, 18 pt, Bold
Formatted: Font: Times New Roman, 18 pt, Bold
Formatted: Font: Times New Roman, 11 pt
Formatted: Font: Times New Roman, 11 pt
Formatted: Font: Times New Roman, 11 pt
Formatted: Font: Times New Roman, 11 pt
Formatted: Line spacing: Double
Formatted: Font: Times New Roman, 11 pt
Formatted: Font: Times New Roman, 11 pt
Formatted: Font: Times New Roman, 11 pt
Formatted: Font: Times New Roman, 11 pt
Formatted: Font: Times New Roman, 11 pt
Formatted: Font: Times New Roman, 11 pt
Formatted: Font: Times New Roman, 11 pt
Formatted: Font: Times New Roman, 11 pt
Formatted: Line spacing: Double
Formatted: Font: Times New Roman, 11 pt
Formatted: Font: Times New Roman, 11 pt
Formatted: Font: Times New Roman, 11 pt
Formatted: Font: Times New Roman, 11 pt
Formatted: Font: Times New Roman, 11 pt
Formatted: Font: Times New Roman, 11 pt
Formatted: Font: Times New Roman, 11 pt
Formatted: Font: Times New Roman, 11 pt
Formatted: Font: Times New Roman, 11 pt
Formatted: Font: Times New Roman, 11 pt
Formatted: Font: Times New Roman, 11 pt
Formatted: Font: Times New Roman, 11 pt
Formatted: Font: Times New Roman, 11 pt
Formatted: Font: Times New Roman, 11 pt
Formatted: Font: Times New Roman, 11 pt

CHINA
Tel:+86-311-85988779
Email: lqx73@163.com

[10] Department of Computer Science, Durham University, Durham, United Kingdom

* Corresponding author

Email: lqx73@163.com (QL)

Abstract~~:~~

Purpose~~:~~

The difference in prognosis between invasive lobular carcinoma (ILC) and invasive ductal carcinoma (IDC) is still controversial in the academic community. Resolving this controversy can help to more accurately determine patients' prognosis, provide further personalized treatment, alleviate unnecessary psychological burden for some patients, and provide direction for further fundamental research.

Patients and ~~Methods:~~ methods

A retrospective cohort study was conducted using the SEER Research Plus Data 8 Registries, Nov 2021 sub (1978-2019), including female breast cancer patients diagnosed with ILC or IDC between 2010 and 2015. Univariate and multivariate Cox regression analyses were performed, and key covariates affecting prognosis were selected. Propensity score matching (PSM) was employed to match patients, and balance tests were conducted to evaluate covariate distribution. Disease-~~Specific Survival~~specific survival (DSS) differences between the matched IDC and ILC groups were compared.

Results~~:~~

Following PSM, the covariate differences between the IDC and ILC groups were significantly reduced. The survival analysis revealed a significantly better prognosis for the IDC group ~~compared to~~than the ILC group (Log-rank test p~~=~~ < 0.~~00019~~001), with a 28.0% increased risk observed in the ILC group.

Conclusion~~:~~

This study provides evidence supporting the existence of significant differences in prognosis

Formatted: Font: Times New Roman, 18 pt
Formatted: Font: Times New Roman, 18 pt
Formatted: Line spacing: Double
Formatted: Font: Times New Roman, 11 pt
Formatted: Font: Times New Roman, 11 pt
Formatted: Line spacing: Double
Formatted: Font: Times New Roman, 11 pt
Formatted: Font: Times New Roman, 11 pt
Formatted: Line spacing: Double
Formatted: Font: Times New Roman, 11 pt
Formatted: Font: Times New Roman, 11 pt
Formatted: Line spacing: Double
Formatted: Font: Times New Roman, 11 pt
Formatted: Font: Times New Roman, 11 pt
Formatted: Font: Times New Roman, 11 pt
Formatted: Font: Times New Roman, 11 pt
Formatted: Font: Times New Roman, 11 pt
Formatted: Line spacing: Double

between IDC and ILC patients after rigorous matching. The IDC group displayed a significantly better prognosis than the ILC group. ~~These~~Notably, these findings have implications for personalized treatment in clinical practice and contribute to the ongoing academic debate on survival differences between IDC and ILC. ~~Further~~However, further research is needed to investigate the biological mechanisms, gene expression, and signaling pathway disparities between IDC and ILC, aiming to provide more targeted guidance for clinical decision-making.

Keywords: ~~IDC;ILC;~~Invasive Ductal Carcinoma, Lobular Carcinomas, Prognosis~~;~~, PSM

Introduction

According to GLOBOCAN 2020 estimates by the International Agency for Research on Cancer, female breast cancer has surpassed lung cancer as the most commonly diagnosed cancer~~.~~ (1). With the incidence of breast cancer increasing ~~year by year~~yearly, it has become one of the significant threats to women's health~~.~~ (2)~~.~~. Invasive lobular carcinoma (ILC) and invasive ductal carcinoma (IDC) are the two most common histological types of breast cancer, with ILC accounting for 10-15% of all breast cancers and IDC accounting for 70-80% of female breast tumors. Despite sharing similar ~~etiology~~etiologies, there are significant differences in biological characteristics, clinical manifestations, and prognosis between ILC and IDC~~.~~ (3-5)~~.~~ ~~Consequently~~. Thus, investigating the differences in Disease-Specific Survival (DSS) among different types of breast cancer patients holds great significance.

There is currently a debate in the academic community regarding the prognostic differences between ILC and IDC (6)~~.~~. In this study, we ~~will use~~used propensity score

Formatted: Font: Times New Roman, 11 pt
Formatted: Font: Times New Roman, 11 pt
Formatted: Font: Times New Roman, 11 pt
Formatted: Line spacing: Double
Formatted: Font: Times New Roman, 11 pt, Bold
Formatted: Font: Times New Roman, 11 pt
Formatted: Font: Times New Roman, 11 pt
Formatted: Font: Times New Roman, 18 pt
Formatted: Justified, Space Before: 0 pt, After: 0 pt
Formatted: Font: Times New Roman, 11 pt
Formatted: Justified, Indent: First line: 2 ch
Formatted: Font: Times New Roman, 11 pt
Formatted: Font: Times New Roman, 11 pt, Font color: Auto
Formatted: Font: Times New Roman, 11 pt
Formatted: Font: Times New Roman, 11 pt
Formatted: Font: Times New Roman, 11 pt
Formatted: Font: Times New Roman, 11 pt
Formatted: Font: Times New Roman, 11 pt
Formatted: Font: Times New Roman, 11 pt
Formatted: Font: Times New Roman, 11 pt
Formatted: Font: Times New Roman, 11 pt
Formatted: Font: Times New Roman, 11 pt
Formatted: Font: Times New Roman, 11 pt
Formatted: Justified, Indent: First line: 2 ch, Widow/Orphan control
Formatted: Font: Times New Roman, 11 pt
Formatted: Font: Times New Roman, 11 pt
Formatted: Font: Times New Roman, 11 pt

matching (PSM) based on generalized linear models to analyze the differences in DSS rates between these two types of breast cancer patients. By comparing and balancing ~~the~~both patient groups' clinical characteristics, molecular subtypes, and treatment methods~~ of both patient groups~~, we aim to reveal potential prognostic differences and provide more accurate prognostic evaluations and treatment recommendations.

PSM is a widely used statistical method in observational studies to eliminate differences between two groups of patients with varying clinical and pathological characteristics, making the study results more reliable ~~,~~(7, 8). Additionally, the PSM method based on generalized linear models can adjust for patient background variables, achieving a balance between comparison groups and reducing bias caused by confounding factors~~. [7, 8]~~(9, 10)~~。~~. Through an in-depth analysis of the prognostic differences between ILC and IDC, we ~~hope~~aim to provide new evidence to resolve this ~~academic~~ controversy and bring new insights to clinical practice, ultimately ~~helping to improve the~~improving breast cancer patients' survival and quality of life ~~of breast cancer patients~~.

Material and methods

Study ~~*Design*~~design and ~~*Population*~~population

The data used in this study were obtained from the Surveillance, Epidemiology, and End Results (SEER) ~~Program, which is~~program, the primary program employed by the National Cancer Institute (NCI) to support cancer surveillance activities in the United States. The SEER ~~Program~~program is an authoritative source of information on cancer incidence and survival ~~and currently~~(11). It collects and publishes cancer incidence and survival data from population-

based cancer registries, covering approximately 48 percent of the U.S. population [https://seer.cancer.gov/registries/cancer_registry/index.html].

After obtaining research permission (Sequence Number: 24787-Nov2021), we utilized SEER*Stat version 8.4.0.1 to retrieve the SEER Research Plus Data 8 Registries. Nov 2021 sub (1978-2019~~), which~~) contains ~~a total of~~ 4,765,822 cases, of which 4,351,209 are malignant cases. Since the patient data provided by SEER is anonymized, ~~and~~ no identifiable information is included in our analysis. Therefore, informed consent was not required.

All female patients included in our analysis had primary tumors ~~located~~ in the breast and were diagnosed with positive histology, confirmed by diagnostic confirmation. The sources of the reports included hospital inpatient, outpatient or clinic, radiation therapy or oncology center (2006 and later), hospital or private laboratory, physician's office/private practice (LMD), nursing/convalescent home/hospice, and other hospital outpatient departments or surgical centers (2006 and later), excluding cases obtained from autopsies and those with only death certificate records. The cause of death (COD) and follow-up/survival months were available as complete dates, and samples with unknown causes of death and follow-up time were excluded. Furthermore, COD to site ~~rec KM~~recode included ~~both~~ alive and breast cancer-specific death patients. We also screened breast cancer patients with ICD-O-3 codes 8500/3: invasive ductal carcinoma and 8520/3: invasive lobular carcinoma.

Formatted: Font: Times New Roman, 11 pt
Formatted: Justified, Indent: First line: 2 ch
Formatted: Font: Times New Roman, 11 pt
Formatted: Font: Times New Roman, 11 pt
Formatted: Font: Times New Roman, 11 pt
Formatted: Font: Times New Roman, 11 pt
Formatted: Font: Times New Roman, 11 pt
Formatted: Font: Times New Roman, 11 pt
Formatted: Justified, Indent: First line: 2 ch
Formatted: Font: Times New Roman, 11 pt
Formatted: Font: Times New Roman, 11 pt
Formatted: Font: Times New Roman, 11 pt
Formatted: Font: Times New Roman, 11 pt
Formatted: Font: Times New Roman, 11 pt

Additionally, we obtained patients' age (<60 years, >=60 years), race (White, Black, Other - including Chinese, Japanese, Vietnamese, Korean, and other Asian ethnicities), marital status (Married, Divorced, Widowed, Single), primary site (Central, Upper Inner, Lower Inner, Upper Outer, Lower Outer, Overlapping, Others), grade (I, II, III, IV), summary stage (Localized, Regional, Distant), AJCC stage (I, II, III, IV), laterality (Right, Left), surgical and radiation sequence (including PORT, NROS, others), systemic surgery sequence (including AAT, NSOST, others), ER/PR status (positive, negative), tumor size (<=1cm, <=2cm, <=3cm, <=4cm, <=5cm, >5cm), and subtype (HR+/HER2+, HR+/HER2-, HR-/HER2+, HR-/HER2-) from the SEER database. ~~A~~Overall, a total of 66,405 patient samples were collected for our analysis.

Statistical ~~*Analysis*~~analysis

We divided the dataset into two groups based on histology: the IDC group and the ILC group~~.~~. To ensure the accuracy and reliability of the analysis, we first used the ks.test function in R version 4.2.1 to perform a Kolmogorov-Smirnov (KS) test on the only continuous variable-survival ~~,~~ to select an appropriate inter-group comparison analysis method. Although the Shapiro-Wilk test is considered a better normality test method, it has higher sensitivity to small sample sizes. When the sample size is large, the Shapiro-Wilk test may result in a misjudgment of P-values~~. Therefore~~ (12). Thus, the KS test is more suitable ~~[9]~~(13)~~。~~ . Based on the results of the KS test (D = 0.053628, p-value < ~~2.2e-16~~0.001), we compared the between-group differences in continuous non-normally distributed variables using the Mann-Whitney U test ~~and~~. Then, we described the sample distribution characteristics of this type of variable using the median (interquartile range~~)~~) ~~[10]~~(14)~~。 For~~. We used the chi-square test for other categorical variables~~, we used the chi-square test~~, which is suitable for large sample sizes, to compare the

Formatted: Font: Times New Roman, 11 pt
Formatted: Justified, Indent: First line: 2 ch
Formatted: Font: Times New Roman, 11 pt
Formatted: Font: Times New Roman, 11 pt
Formatted: Font: Times New Roman, 16 pt, Not Italic
Formatted: Justified, Widow/Orphan control
Formatted: Font: Times New Roman, 16 pt, Not Italic
Formatted: Font: Times New Roman, 11 pt
Formatted: Justified, Indent: First line: 2 ch, Widow/Orphan control
Formatted: Font: Times New Roman, 11 pt
Formatted: Font: Times New Roman, 11 pt
Formatted: Font: Times New Roman, 11 pt
Formatted: Font: Times New Roman, 11 pt
Formatted: Font: Times New Roman, 11 pt
Formatted: Font: Times New Roman, 11 pt
Formatted: Font: Times New Roman, 11 pt
Formatted: Font: Times New Roman, 11 pt
Formatted: Font: Times New Roman, 11 pt
Formatted: Font: Times New Roman, 11 pt
Formatted: Font: Times New Roman, 11 pt

distribution differences between the IDC and ILC groups. ~~We~~Moreover, we calculated the corresponding P-values and described the distribution of samples within each group using frequency (percentage~~)[11]。 Through this method, we were able to~~) (15). Through this method, we could fully understand the differences in various variables between the IDC and ILC groups, providing a strong basis for the subsequent analysis using the PSM method based on generalized linear models.

Before PSM, we considered the presence of confounding factors. ~~In order to~~To reduce bias risks and achieve better~~-~~matching results, we identified important variables ~~that affect~~affecting prognosis through Cox regression analysis ~~and~~. We included them as input variables in the PSM model. Specifically, we used the coxph function of the survival package to perform univariate Cox regression analysis for the IDC and ILC groups, respectively, and included covariates with significant statistical differences ($P < 0.$~~05~~050) in subsequent multivariate Cox regression analysis. Based on the potential interaction between covariates, we further estimated the independent prognostic ability of each variable. We then evaluated the relationship between each variable and survival by calculating the hazard ratio, 95% confidence interval, and P-value for both univariate and multivariate variable analyses. ~~We~~Furthermore, we excluded variables with P-values greater than 0.~~05~~050 in ~~both~~ univariate and multivariate Cox regression analysis results for ~~both~~ the IDC and ILC groups~~, and~~. We also used the remaining variables as covariates closely related to survival for subsequent PSM to minimize the weakening of validity and unnecessary sample loss brought about by including too many irrelevant variables.

Formatted: Font: Times New Roman, 11 pt

Formatted: Font: Times New Roman, 11 pt

Formatted: Font: Times New Roman, 11 pt

Formatted: Justified, Indent: First line: 2 ch

Formatted: Font: Times New Roman, 11 pt

Formatted: Font: Times New Roman, 11 pt

Formatted: Font: Times New Roman, 11 pt

Formatted: Font: Times New Roman, 11 pt

Formatted: Font: Times New Roman, 11 pt

Formatted: Font: Times New Roman, 11 pt

Formatted: Font: Times New Roman, 11 pt

Formatted: Font: Times New Roman, 11 pt

Formatted: Font: Times New Roman, 11 pt

Formatted: Font: Times New Roman, 11 pt

Formatted: Font: Times New Roman, 11 pt

Formatted: Font: Times New Roman, 11 pt

Formatted: Font: Times New Roman, 11 pt

~~Afterwards~~Afterward, we constructed a binary logistic regression model with ~~Histology~~histology as the dependent variable and the covariates identified from the Cox regression analysis as independent variables using the generalized linear model (GLM) function of the matchit package. The propensity score ~~(PS)~~ value, representing the probability of each individual being assigned to the IDC or ILC group, was calculated for each individual. Then, using the nearest neighbor matching algorithm with a caliper matching threshold of 0.0001 and a 1:1 ratio, we matched IDC and ILC individuals based on their propensity scores~~,~~ and randomized the matching order to improve matching precision and reduce confounding bias~~, thus improving~~. Thus, this improves the quality of ~~the comparison between~~comparing the two ~~different~~ pathological types regarding the difference in prognosis and risk. ~~Subsequently~~To further screen the variables that had a more significant impact on the grouping, we subsequently used the love.plot function of the cobalt package to conduct a balance test after matching, setting a threshold of 0.05~~, to further screen~~. We repeated the ~~variables that had a greater impact on the grouping. We compared the~~comparison of differences between different variables in the IDC and ILC groups ~~again, and~~. After matching, we plotted the baseline table of clinical data ~~after matching~~ to verify whether the differences between the groups were effectively controlled and ensure the reliability of the study results. Finally, we compared the prognosis differences between the matched IDC and ILC groups using the survival function of the R package survival~~,~~ ~~and~~. Furthermore, we validated the significance of the differences in prognosis between the two groups using three statistical methods: the log-rank test, the Wald test, and the likelihood ratio test.

Formatted: Justified, Indent: First line: 2 ch
Formatted: Font: Times New Roman, 11 pt
Formatted: Font: Times New Roman, 11 pt
Formatted: Font: Times New Roman, 11 pt
Formatted: Font: Times New Roman, 11 pt
Formatted: Font: Times New Roman, 11 pt
Formatted: Font: Times New Roman, 11 pt
Formatted: Font: Times New Roman, 11 pt
Formatted: Font: Times New Roman, 11 pt
Formatted: Font: Times New Roman, 11 pt
Formatted: Font: Times New Roman, 11 pt
Formatted: Font: Times New Roman, 11 pt
Formatted: Font: Times New Roman, 11 pt
Formatted: Font: Times New Roman, 11 pt
Formatted: Font: Times New Roman, 11 pt
Formatted: Font: Times New Roman, 11 pt
Formatted: Font: Times New Roman, 11 pt

Results

We obtained 510,385 ~~samples of~~ primary breast cancer samples from the SEER Research Plus Data 8 Registries. Nov 2021 ~~s-ub~~sub (1978-2019), of which 66,405 female breast cancer patients were included in our analysis after excluding and including samples according to our criteria. Of these, 58,497 cases were IDC~~)~~((88.~~09%)~~1%), and 7,908 ~~cases~~ were ILC (11.~~91%). There~~9%). Additionally, there were 5,726 cases of breast cancer-specific deaths (9.~~91~~9%) in the IDC group and 853 cases (10.~~79~~8%) in the ILC group. The median survival time in the IDC group was 75.~~000 [~~0 (58.~~000~~0, 95.~~000]~~0) months, while in the ILC group, it was 73.~~000 [~~0 (57.~~000~~0, 93.~~000]~~0) months. ~~The~~Moreover, the distributions of age, race, marital status, primary tumor site, molecular subtype, pathological grade, summary stage, AJCC, order of surgery and systemic therapy, order of surgery and radiation therapy, hormone status, and tumor size were statistically significantly different between the IDC and ILC groups. Notably, the proportion of elderly patients in the ILC group (61.~~06~~1%) was significantly higher than that in the IDC group (50.~~76%). In terms of~~8%). Regarding molecular subtypes, the HR+/HER2- subtype accounted for ~~93.98~~94.0% of the ILC group~~, while~~. In contrast, it was only 72.0% in the IDC group~~, it was only 71.94%,~~ and the other three subtypes were lower in proportion in the ILC group than in the IDC group. In terms of pathological grade, the proportion of Grade I (31.~~55~~6%) and Grade II (61.~~33~~3%) in the ILC group was significantly higher than that in the IDC group (23.~~38~~4% and 41.~~45~~5%, respectively~~), while~~). In comparison, the proportion of Grade III (35.~~03~~0%) and Grade IV (0.~~13~~1%) in the IDC group was significantly higher than that in the ILC group (Grade III: 7.~~08~~1%, Grade IV: 0.~~04%). Additionally, in terms of hormone status~~0%). Furthermore, the proportions of ER-positive (98.~~05~~1%) and PR-positive (83.~~54~~5%)

Formatted: Font: Times New Roman, 18 pt
Formatted: Justified, Space Before: 0 pt, After: 0 pt
Formatted: Font: Times New Roman, 11 pt
Formatted: Justified, Indent: First line: 2 ch, Widow/Orphan control
Formatted: Font: Times New Roman, 11 pt
Formatted: Font: Times New Roman, 11 pt
Formatted: Font: Times New Roman, 11 pt
Formatted: Font: Times New Roman, 11 pt
Formatted: Font: Times New Roman, 11 pt
Formatted: Font: Times New Roman, 11 pt
Formatted: Font: Times New Roman, 11 pt
Formatted: Font: Times New Roman, 11 pt
Formatted: Font: Times New Roman, 11 pt
Formatted: Font: Times New Roman, 11 pt
Formatted: Font: Times New Roman, 11 pt
Formatted: Font: Times New Roman, 11 pt
Formatted: Font: Times New Roman, 11 pt
Formatted: Font: Times New Roman, 11 pt
Formatted: Font: Times New Roman, 11 pt
Formatted: Font: Times New Roman, 11 pt
Formatted: Font: Times New Roman, 11 pt
Formatted: Font: Times New Roman, 11 pt
Formatted: Font: Times New Roman, 11 pt
Formatted: Font: Times New Roman, 11 pt
Formatted: Font: Times New Roman, 11 pt
Formatted: Font: Times New Roman, 11 pt
Formatted: Font: Times New Roman, 11 pt
Formatted: Font: Times New Roman, 11 pt
Formatted: Font: Times New Roman, 11 pt
Formatted: Font: Times New Roman, 11 pt
Formatted: Font: Times New Roman, 11 pt
Formatted: Font: Times New Roman, 11 pt
Formatted: Font: Times New Roman, 11 pt
Formatted: Font: Times New Roman, 11 pt
Formatted: Font: Times New Roman, 11 pt
Formatted: Font: Times New Roman, 11 pt
Formatted: Font: Times New Roman, 11 pt
Formatted: Font: Times New Roman, 11 pt

patients in the ILC group were significantly higher than those in the IDC group (ER: 82.~~24~~2%, PR: 71.~~83%). In~~8%) in terms of hormone status. For tumor size, the proportion of tumors with a diameter of 2cm or less was higher in the IDC group than in the ILC group (64.~~82~~8% vs. 51.~~04%), while~~0%). In comparison, the proportion of tumors with a diameter of 2cm or more in the ILC group was higher than that in the IDC group. (Table 1).

~~Table1:~~ Table 1. Clinical data Baseline table before propensity score matching

level	Overall	IDC	ILC	p
n	66405	58497	7908	
survival (median [IQR])	75.~~00~~0 [57.~~00~~0, 95.~~00~~0]	75.~~00~~0 [58.~~00~~0, 95.~~00~~0]	73.~~00~~0 [57.~~00~~0, 93.~~00~~0]	<0.~~0001~~001
status (%)				
Alive	59826 (90.~~09~~1)	52771 (90.~~21~~2)	7055 (89.~~21~~2)	0.~~0056~~006
Dead	6579 (9.~~91~~9)	5726 (9.~~79~~8)	853 (10.~~79~~8)	
age (%)				
<60	31885 (48.~~02~~0)	28806 (49.~~24~~2)	3079 (38.~~94~~9)	<0.~~0001~~001
>=60	34520 (~~51.98~~52.0)	29691 (50.~~76~~8)	4829 (61.~~06~~1)	
race (%)				
White	55083 (~~82.95~~83.0)	48103 (82.~~23~~2)	6980 (88.~~27~~3)	<0.~~0001~~001
Black	5879 (8.~~85~~9)	5339 (9.~~13~~1)	540 (6.~~83~~8)	
Other	5443 (8.~~20~~2)	5055 (8.~~64~~6)	388 (4.~~91~~9)	
Maritial status (%)				
Married	40847 (61.~~51~~5)	35952 (61.~~46~~5)	4895 (61.~~90~~9)	<0.~~0001~~001
Divorced	7807 (11.~~76~~8)	6880 (11.~~76~~8)	927 (11.~~72~~7)	
Widowed	7308 (11.~~01~~0)	6299 (10.~~77~~8)	1009 (12.~~76~~8)	
single	10443 (15.~~73~~7)	9366 (16.~~01~~0)	1077 (13.~~62~~6)	
Primary Site (%)				
Central	2755 (4.~~15~~2)	2376 (4.~~06~~1)	379 (4.~~79~~8)	<0.~~0001~~001
Upper inner	8704 (13.~~11~~1)	7869 (13.~~45~~5)	835 (10.~~56~~6)	

Lower inner	3787 (5.~~70~~7)	3466 (5.~~93~~9)	321 (4.~~06~~1)	
Upper outer	23034 (34.~~69~~7)	20256 (34.~~63~~6)	2778 (35.~~13~~1)	
Lower outer	5270 (7.~~94~~9)	4659 (~~7.96~~8.0)	611 (7.~~73~~7)	
Overlapping	15453 (23.~~27~~3)	13621 (23.~~28~~3)	1832 (23.~~17~~2)	
others	7402 (11.~~15~~2)	6250 (10.~~68~~7)	1152 (14.~~57~~6)	
Subtype (%)				
HR+/HER2+	7020 (10.~~57~~6)	6687 (11.~~43~~4)	333 (4.~~21~~2)	<0.~~0001~~001
HR+/HER2-	49513 (74.~~56~~6)	42081 (~~71.94~~72.0)	7432 (~~93.98~~94.0)	
HR-/HER2+	2844 (4.~~28~~3)	2807 (4.~~80~~8)	37 (0.~~47~~5)	
HR-/HER2-	7028 (10.~~58~~6)	6922 (11.~~83~~8)	106 (1.~~34~~3)	
Grade (%)				
I	16173 (24.~~36~~4)	13678 (23.~~38~~4)	2495 (31.~~55~~6)	<0.~~0001~~001
II	29097 (43.~~82~~8)	24247 (41.~~45~~5)	4850 (61.~~33~~3)	
III	21054 (31.~~71~~7)	20494 (35.~~03~~0)	560 (7.~~08~~1)	
IV	81 (0.~~12~~1)	78 (0.~~13~~1)	3 (0.~~04~~0)	
Summary stage (%)				
Localized	44707 (67.~~32~~3)	39655 (67.~~79~~8)	5052 (63.~~88~~9)	<0.~~0001~~001
Regional	19241 (~~28.98~~29.0)	16713 (28.~~57~~6)	2528 (~~31.97~~32.0)	
Distant	2457 (3.~~70~~7)	2129 (3.~~64~~6)	328 (4.~~15~~2)	
AJCC (%)				
I	36096 (54.~~36~~4)	32596 (55.~~72~~7)	3500 (44.~~26~~3)	<0.~~0001~~001
II	21498 (32.~~37~~4)	18585 (31.~~77~~8)	2913 (36.~~84~~8)	
III	6458 (9.~~73~~7)	5280 (9.~~03~~0)	1178 (14.~~90~~9)	
IV	2353 (3.~~54~~5)	2036 (3.~~48~~5)	317 (4.~~01~~0)	
Laterality (%)				
Right	32886 (49.~~52~~5)	29037 (49.~~64~~6)	3849 (48.~~67~~7)	0.~~1094~~109
Left	33519 (50.~~48~~5)	29460 (50.~~36~~4)	4059 (51.~~33~~3)	
Systemic Sur Seq (%)				
AAT	45514 (68.~~54~~5)	39718 (67.~~90~~9)	5796 (73.~~29~~3)	<0.~~0001~~001
NSOST	13284 (20.~~00~~0)	11795 (20.~~16~~2)	1489 (18.~~83~~8)	

others	7607 (11.465)	6984 (11.949)	623 (7.889)	
Surg Rad Seq (%)				
PORT	37712 (56.798)	33461 (57.202)	4251 (53.768)	<0.0001001
NROS	27921 (42.951)	24319 (41.576)	3602 (45.556)	
others	772 (1.162)	717 (1.232)	55 (0.707)	
ER (%)				
Positive	55861 (84.121)	48107 (82.242)	7754 (98.051)	<0.0001001
Negtive	10544 (15.889)	10390 (17.768)	154 (1.952.0)	
PR (%)				
Positive	48625 (73.222)	42019 (71.838)	6606 (83.545)	<0.0001001
Negtive	17780 (26.788)	16478 (28.172)	1302 (16.465)	
Tumor size (%)				
<=1	18397 (27.707)	16822 (28.768)	1575 (19.9220.0)	<0.0001001
<=2	23557 (35.475)	21096 (36.061)	2461 (31.121)	
<=3	12165 (18.323)	10656 (18.222)	1509 (19.081)	
<=4	5000 (7.535)	4320 (7.384)	680 (8.606)	
<=5	2559 (3.859)	2092 (3.586)	467 (5.919)	
>5	4727 (7.121)	3511 (6.000)	1216 (15.384)	

Abbreviation: AAT, Adjuvant Therapy; NSOST, No systemic therapy and/or surgical therapy; PORT, Post-Operative Radiation Therapy; NROS, No radiation and/or cancer-directed surgery.

The results of univariate and multivariate Cox regression analysis results of IDC and ILC patients are summarized in Tables S1 and S2. In Tables. From the univariate Cox regression analysis of IDC, Lateralitylaterality showed no significant independent prognostic ability (P=0.747), and the independent prognostic efficacy of the others subcategoryother subcategories in the surgery and radiation therapy sequence was not significantinsignificant

(P=0.143). ~~In its~~From the multivariate Cox regression analysis, none of the seven Primary_site prognostic abilities were significant, and the prognostic ability of the HR-/HER2- subtype ~~in Subetype~~ was ~~not significant~~insignificant (P=0.~~7428~~743). The prognostic efficacy of the ~~others subcategory~~other subcategories in the surgery and radiation therapy sequence was ~~not significant~~also insignificant (P=0.~~2602~~260) (S1 Table).

~~In~~From the univariate Cox regression analysis of ILC, the independent prognostic efficacy of the ~~others subcategory~~other subcategories in Primary_site was ~~not significant~~insignificant (P=0.263~~),~~). Laterality showed no significant independent prognostic ability (P=0.633), and the independent prognostic efficacy of the HR+/HER2- subtype ~~in Subetype~~ was ~~not significant~~insignificant (P=0.118). ~~In~~From the multivariate Cox regression analysis of ILC, Black patients and others showed no significant prognostic ability compared to White~~, the~~ patients. The single marital status showed no significant prognostic efficacy (P=0.~~49), and consistent with the multiple regression analysis of IDC,~~ 490). Additionally, none of the seven different Primary site prognostic abilities were significant~~.~~, consistent with the multiple regression analysis of IDC. The prognostic efficacy of the ~~others subcategory~~other subcategories in the surgery and radiation therapy sequence in ILC was ~~not significant~~insignificant (P=0.~~7796~~780), and the prognostic efficacy of the HR+/HER2- subtype ~~in Subetype~~ was ~~not significant~~insignificant (P=0.~~1989). Tumor~~199). Furthermore, tumor size between 2 and 3 cm~~, as well as~~ and greater than 5 cm~~,~~ did not show a clear prognostic association compared to a tumor size of 1 cm~~.(~~ (S2 Table).

Additionally, theThe prognostic ability of Grade IV was not significantinsignificant in both univariate and multivariate Cox regression analysis results, but. Still, we consider this to be due to the small number of Grade IV samples in ILC, rather than its true situation. Finally, we included variables other than Lateralitylaterality that had no significant prognostic ability in both univariate and multivariate Cox regression results in the calculation of the propensity score.

In the balance test of covariates before and after PSM, weWe first calculated the absolute standardized mean difference (AMD) of each covariate from the balance test of covariates before and after PSM. Notably, AMD is a measure for evaluating the degree of distributional differences of covariates before and after matching[12], and the absolute mean difference graph of covariates was plotted (Figure 1, left panel)。 (16), and the absolute mean difference graph of covariates was plotted (Fig 1, left panel). It can be seen from the graph that the absolute standardized mean differences between each covariate of IDC and ILC groups were all less than 0.05050 after PSM, indicatingsuggesting that the differences between each covariate in the two groups were significantly reduced after matching. Moreover, in the baseline data distribution after PSM (Table 2), there were no significant differences observed in the distribution of all variables related to DSS between IDC and ILC groups (P>0.05).050). However, before PSM, the absolute standardized mean differences of covariates such as age, AJCC stage, Gradegrade classification, ER and PR status, molecular subtypes, and tumor size were all greater than 0.05 before PSM, indicating that there were significant differences in thethese covariates' distribution of these covariates between the two groups.

Figure 1. Covariate Balance Test Plot after Propensity Score Matching.

Formatted: Justified, Indent: First line: 2 ch, Widow/Orphan control
Formatted: Font: Times New Roman, 11 pt
Formatted: Font: Times New Roman, 11 pt
Formatted: Font: Times New Roman, 11 pt
Formatted: Font: Times New Roman, 11 pt
Formatted: Font: Times New Roman, 11 pt
Formatted: Font: Times New Roman, 11 pt
Formatted: Justified, Indent: First line: 2 ch, Widow/Orphan control
Formatted: Font: Times New Roman, 11 pt
Formatted: Font: Times New Roman, 11 pt
Formatted: Font: Times New Roman, 11 pt
Formatted: Font: Times New Roman, 11 pt
Formatted: Font: Times New Roman, 11 pt
Formatted: Font: Times New Roman, 11 pt
Formatted: Font: Times New Roman, 11 pt
Formatted: Font: Times New Roman, 11 pt
Formatted: Font: Times New Roman, 11 pt
Formatted: Font: Times New Roman, 11 pt
Formatted: Font: Times New Roman, 11 pt
Formatted: Font: Times New Roman, 11 pt
Formatted: Font: Times New Roman, 11 pt

Table2 : ClinicalTable 2. Baseline clinical data baseline table after propensity score matching.

Characteristic	Overall	IDC	ILC	p
NO.	12794	6397	6397	
survival (median [IQR])	75.000 [58.0000, 95.0000]	76.000 [59.0000, 96.0000]	75.000 [58.0000, 94.0000]	0.0051005
status (%)				
Alive	11821 (92.394)	5944 (92.929)	5877 (91.879)	0.0277028
Dead	973 (7.616)	453 (7.081)	520 (8.131)	
age (%)				
<60	5216 (40.778)	2625 (41.030)	2591 (40.505)	0.5527553
>=60	7578 (59.232)	3772 (58.9759.0)	3806 (59.505)	
race (%)				
Black	690 (5.394)	344 (5.384)	346 (5.414)	0.9755976
Other	633 (4.955.0)	314 (4.919)	319 (4.995.0)	
White	11471 (89.666)	5739 (89.717)	5732 (89.606)	
Maritial status (%)				
Divorced	1385 (10.838)	699 (10.939)	686 (10.727)	0.979
Married	8283 (64.747)	4140 (64.727)	4143 (64.768)	
single	1654 (12.939)	827 (12.939)	827 (12.939)	
Widowed	1472 (11.515)	731 (11.434)	741 (11.586)	
Primary Site (%)				
Central	516 (4.030)	261 (4.081)	255 (3.994.0)	0.9985999
Lower outer	962 (7.525)	481 (7.525)	481 (7.525)	
Lower inner	508 (3.974.0)	256 (4.00)	252 (3.949)	
others	1675 (13.091)	826 (12.919)	849 (13.273)	
Overlapping	2996 (23.424)	1502 (23.485)	1494 (23.354)	
Upper outer	4733 (36.9937.0)	2368 (37.020)	2365 (36.9737.0)	
Upper inner	1404 (10.9711.0)	703 (10.9911.0)	701 (10.9611.0)	
Subtype (%)				

HR-/HER2-	181 (1.414)	85 (1.333)	96 (1.505)	0.7105711
HR-/HER2+	66 (0.525)	34 (0.535)	32 (0.505)	
HR+/HER2-	11984 (93.677)	5987 (93.596)	5997 (93.758)	
HR+/HER2+	563 (4.404)	291 (4.556)	272 (4.253)	
Grade (%)				
I	3725 (29.121)	1833 (28.657)	1892 (29.586)	0.4121412
II	7977 (62.354)	4004 (62.596)	3973 (62.111)	
III	1092 (8.545)	560 (8.758)	532 (8.323)	
AJCC (%)				
I	6833 (53.414)	3427 (53.576)	3406 (53.242)	0.4400
II	4354 (34.030)	2186 (34.172)	2168 (33.899)	
III	1279 (10.000)	635 (9.939)	644 (10.071)	
IV	328 (2.56 6)	149 (2.333)	179 (2.808)	
Laterality (%)				
Left	6372 (49.808)	3066 (47.939)	3306 (51.687)	<0.0001001
Right	6422 (50.202)	3331 (52.071)	3091 (48.323)	
Systemic Sur Seq (%)				
AAT	9643 (75.374)	4846 (75.758)	4797 (74.9975.0)	0.5883588
NSOST	2402 (18.778)	1185 (18.525)	1217 (19.020)	
others	749 (5.859)	366 (5.727)	383 (5.996.0)	
Surg Rad Seq (%)				
NROS	5732 (44.808)	2860 (44.717)	2872 (44.909)	0.5409541
others	71 (0.556)	31 (0.485)	40 (0.636)	
PORT	6991 (54.646)	3506 (54.818)	3485 (54.485)	
ER (%)				
Negtive	266 (2.081)	127 (1.992.0)	139 (2.172)	0.4955496
Positive	12528 (97.929)	6270 (98.010)	6258 (97.838)	
PR (%)				
Negtive	1669 (13.056)	836 (13.071)	833 (13.020)	0.9581958
Positive	11125 (86.9587.0)	5561 (86.939)	5564 (86.9887.0)	

Tumor size (%)				
<=1	3041 (23.~~77~~8)	1514 (23.~~67~~7)	1527 (23.~~87~~9)	0.937
<=2	4655 (36.~~38~~4)	2317 (36.~~22~~2)	2338 (36.~~55~~6)	
<=3	2622 (20.~~49~~5)	1323 (20.~~68~~7)	1299 (20.~~31~~3)	
<=4	944 (7.~~38~~4)	464 (7.~~25~~3)	480 (7.~~50~~5)	
<=5	470 (3.~~67~~7)	243 (3.~~80~~8)	227 (3.~~55~~6)	
>5	1062 (8.~~30~~3)	536 (8.~~38~~4)	526 (8.~~22~~2)	

Abbreviation: AAT, Adjuvant Therapy; NSOST, No systemic therapy and/or surgical therapy; PORT, Post-Operative Radiation Therapy; NROS, No radiation and/or cancer-directed surgery.

Next, we analyzed the differences between each covariate in the cumulative distribution functions~~, i.e.,~~ and applied the KS statistic. The KS statistic is a non-parametric test method ~~used~~ to measure the difference between two distributions. It is calculated as the maximum distance between the cumulative distribution functions of the two distributions~~.~~ (17). In this study, we calculated the KS statistic for each covariate before and after matching (~~Figure~~Fig 1, right panel). ~~Before PSM, the~~The KS statistic of covariates such as age, AJCC stage, ~~Grade~~grade classification, ER and PR status, molecular subtypes, and tumor size were all greater than 0.05 before PSM, indicating that the distribution of these covariates within the sample before matching was unbalanced. However, after PSM, the KS statistic of each covariate was less than 0.05, almost approaching 0, consistent with the distribution of absolute mean difference, ~~indicating~~implying that the sample was balanced on these covariates after matching.

Fig 1. Covariate Balance Test Plot after Propensity Score Matching.

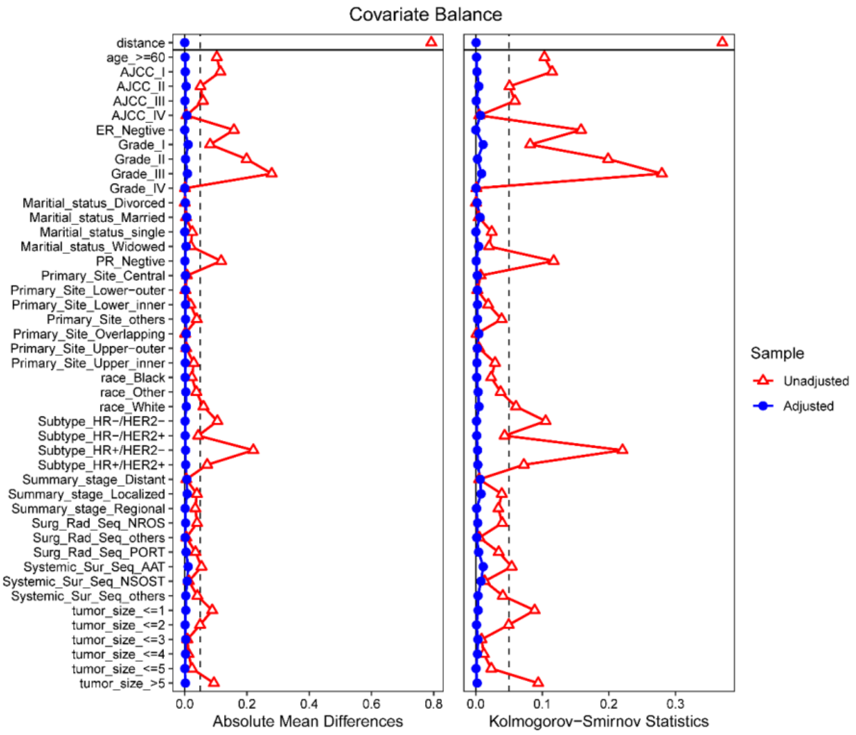

Finally, we conducted a survival analysis on the matched IDC and ILC groups. The KM survival analysis results showed that the prognosis of the IDC group was significantly better than that of the ILC group (Log-rank test p~~=~~ < 0.~~00019)(Figure~~001) (Fig 2). Compared with the IDC group, Cox proportional hazards regression analysis showed that the risk of the ILC group ~~increased by~~was 28~~%.~~.0% higher than the IDC group (HR=1.28, CI=1.12-1.45). Meanwhile, the results of the ~~Likelihood~~likelihood ratio test (W value: 1.96, p~~=2e-04~~ < 0.001) and Wald test (W value: 11.87, p=~~2e-04~~ < 0.001) were consistent with the Log-rank test results, further confirming the significant difference in prognosis between the IDC and ILC groups.

~~Figure~~Fig 2. KM ~~survival~~Survival analysis of IDC and ILC groups after PSM.

Formatted: Font: Times New Roman, 11 pt
Formatted: Justified, Indent: First line: 2 ch, Widow/Orphan control
Formatted: Font: Times New Roman, 11 pt
Formatted: Font: Times New Roman, 11 pt
Formatted: Font: Times New Roman, 11 pt
Formatted: Font: Times New Roman, 11 pt
Formatted: Font: Times New Roman, 11 pt
Formatted: Font: Times New Roman, 11 pt
Formatted: Font: Times New Roman, 11 pt
Formatted: Font: Times New Roman, 11 pt
Formatted: Font: Times New Roman, 11 pt
Formatted: Justified
Formatted: Font: Times New Roman, 11 pt
Formatted: Font: Times New Roman, 11 pt, Bold
Formatted: Font: Times New Roman, 11 pt, Bold

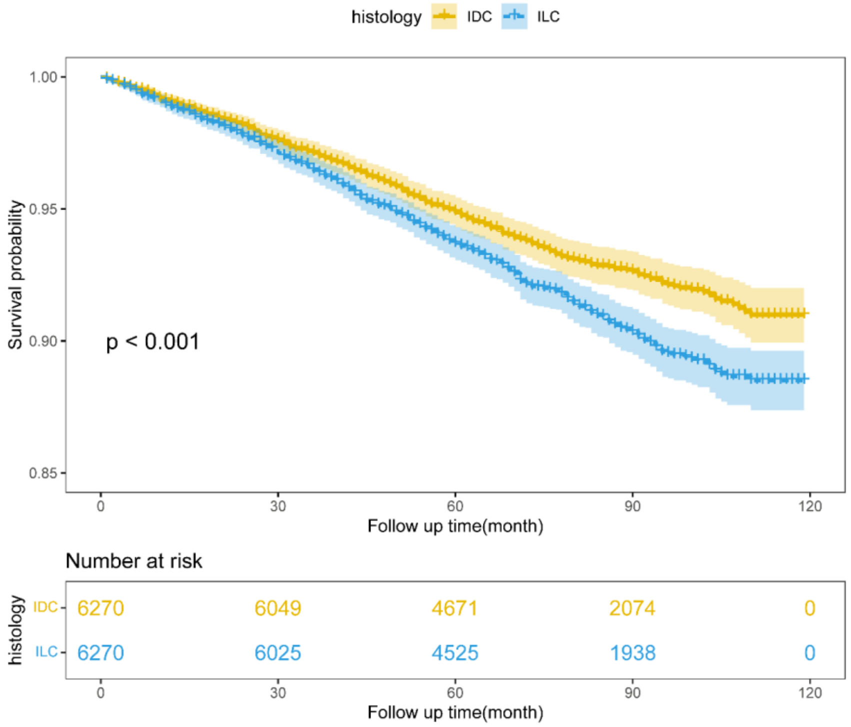

Discussion

IDC is the most prevalent breast cancer type, believed to primarily originate from malignant epithelial cells of the breast's terminal ducts [13]。 (18). IDC is characterized by invasive growth, with tumor cells infiltrating the breast stroma and spreading after breaking through ductal walls. Microscopically, tumor cells often form cord-like, cluster-like, or small beam-like patterns. Clinically, IDC manifests mainly as a nipple-like and solid mass. IILC, the second most common invasive breast cancer histological type after IDC, originates primarily from malignant cells beneath the breast lobule's epithelium. (19). Microscopically, ILC often exhibits small, uniform, and non-adhesive cancer cells distributed individually or diffusely in

Formatted: Font: Times New Roman, 11 pt

Formatted: Font: Times New Roman, 18 pt

Formatted: Justified, Space Before: 0 pt, After: 0 pt

Formatted: Font: Times New Roman, 11 pt

Formatted: Justified, Indent: First line: 2 ch, Widow/Orphan control

Formatted: Font: Times New Roman, 11 pt

Formatted: Font: Times New Roman, 11 pt

the fibrous stroma ~~[14]~~(20)~~。~~. Compared to IDC, ILC lacks ~~obvious~~apparent clinical masses ~~clinically~~ and has a lower detection rate on breast ultrasound and X-ray examinations ~~[15]。~~ (21).

Besides the aforementioned histological differences, ILC and IDC also display molecular disparities. ILC patients lack E-cadherin expression, a deficiency that stimulates the activation signal of Epithelial-Mesenchymal Transition (EMT), resulting in a higher incidence of bone, gastrointestinal, omental, and ovarian metastases compared to IDC patients ~~[16-18]~~(22-25)~~。~~. Moreover, studies have revealed that genomic profiles of ILC and IDC patients differ based on tissue sequencing assessments. ILC patients exhibit a higher tumor mutation burden~~, as well as~~ and variations in immune infiltration and copy number changes~~[19-22]~~(26-29)~~。 Despite~~. Furthermore, even though the ~~evident histological and genomic differences between IDC and ILC,~~ clinical ~~treatments~~treatment methods for both ~~remain~~are relatively similar~~.~~, some differences still exist. For instance, in clinical practice, the frequency of ILC patients undergoing mastectomy is slightly higher than that of IDC patients (24). Furthermore, there is ongoing debate regarding the clinical outcomes of ILC and IDC patients. Wasif et al. discovered that ILC patients had a significantly higher five-year DSS rate compared to IDC patients (P < 0.001) through stage-matched analysis~~[23]。 Chen et al.~~(30). Chen et al. obtained similar results, with ILC patients having a survival advantage within five years post-surgery compared to IDC patients~~,~~ but demonstrating a worse long-term prognosis [24]. Furthermore, Yang et al. found no significant difference in overall survival rates between ILC and IDC patients during the first five years after PMS analysis [25]. In contrast, some studies have reported that ILC patients have a higher ten-year overall survival rate ~~compared to~~than IDC patients~~[24, 25]~~(31, 32)~~。~~.

Formatted: Font: Times New Roman, 11 pt
Formatted: Font: Times New Roman, 11 pt
Formatted: Font: Times New Roman, 11 pt
Formatted: Font: Times New Roman, 11 pt
Formatted: Font: Times New Roman, 11 pt
Formatted: Font: Times New Roman, 11 pt
Formatted: Font: Times New Roman, 11 pt
Formatted: Font: Times New Roman, 11 pt
Formatted: Font: Times New Roman, 11 pt
Formatted: Font: Times New Roman, 11 pt
Formatted: Font: Times New Roman, 11 pt
Formatted: Font: Times New Roman, 11 pt
Formatted: Font: Times New Roman, 11 pt
Formatted: Font: Times New Roman, 11 pt
Formatted: Font: Times New Roman, 11 pt
Formatted: Font: Times New Roman, 11 pt
Formatted: Font: Times New Roman, 11 pt
Formatted: Font: Times New Roman, 11 pt
Formatted: Font: Times New Roman, 11 pt
Formatted: Font: Times New Roman, 11 pt
Formatted: Font: Times New Roman, 11 pt
Formatted: Font: Times New Roman, 11 pt

Considering the ~~aforementioned~~abovementioned research ~~findings~~, we hypothesize that the differences in study outcomes may stem from variations in the chosen study populations, variable control, and distinct data analysis methods. ~~Consequently~~Therefore, to provide more comprehensive and precise data, this study ~~employed PSM to meticulously match IDC and ILC patients~~matched with a more comprehensive set of covariates and their subclasses, eliminating the impact of baseline patient characteristics. Using PSM based on a generalized linear model, we explored the disparities in DSS between these two types of breast cancer patients. Through balancing tests of covariates before and after matching, the disparities in various covariates between the IDC and ILC groups were significantly reduced, offering a more balanced foundation for subsequent survival analysis. ~~In~~From the survival analysis post-PSM, we discovered that the prognosis of the IDC group was significantly better than that of the ILC group (Log-rank test p~~=~~ < 0.~~00019~~001), and the risk of the ILC group increased by 28.0% compared to the IDC group. This result indicates that after PSM, the difference in tumor-specific survival between IDC and ILC patients is substantial, ~~further supporting the notion that IDC and ILC have distinct clinical outcomes. This study's advantage lies in the use of PSM to effectively eliminate the influence of baseline patient characteristics on the study results, rendering the comparison between IDC and ILC patients more objective.~~supporting the notion that IDC and ILC have distinct clinical outcomes. In contrast, a recent study conducted by Ciqiu Yang et al. showed that prior to PSM, the overall survival (OS) of IDC patients was superior to that of ILC patients (HR=1.045, P=0.025, 95%CI: 1.007-1.085). However, after PSM, the difference in OS between the two groups disappeared. In addition to the reasons for these divergent results mentioned above, the differing focus outcomes may be one of the leading

Formatted: Font: Times New Roman, 11 pt

Formatted: Font: Times New Roman, 11 pt

Formatted: Font: Times New Roman, 11 pt

Formatted: Font: Times New Roman, 11 pt

Formatted: Font: Times New Roman, 11 pt

Formatted: Font: Times New Roman, 11 pt

Formatted: Font: Times New Roman, 11 pt

Formatted: Font: Times New Roman, 11 pt

Formatted: Font: Times New Roman, 11 pt

causes of such discrepancies. For breast cancer patients with longer survival periods, DSS can help us avoid the influences of other potential interfering factors more effectively than OS, thereby reflecting the impact of breast cancer on survival more accurately.

This study ~~still~~ has some noted limitations. As a retrospective study, the data is sourced from the SEER database. Although this is an authoritative large-scale cancer registry database, its population coverage ~~is~~ primarily ~~composed of~~comprises white individuals in the United States. To further verify the universality and representativeness of these conclusions, it is necessary to expand the sample sources and scope for patients from other regions and ethnicities. Additionally, although we have substantially reduced biases through PSM, there may still be unobserved potential confounding factors that could affect the accuracy of the results.

Conclusion

Through ~~the application of~~applying PSM, our study discovered that, upon stringent matching, the prognosis for patients in the IDC group was markedly superior to those in the ILC group. This finding offers compelling evidence to support individualized treatment strategies for patients with IDC and ILC in clinical settings. Nevertheless, it remains essential for future research to delve deeper into the distinctions between IDC and ILC concerning biological mechanisms, gene expression patterns, and signaling pathways~~, with the aim of providing~~ to provide more precise and targeted guidance in clinical practice.

Acknowledgments

Formatted: Font: Times New Roman, 11 pt

Formatted: Font: Times New Roman, 11 pt

Formatted: Font: Times New Roman, 11 pt

Formatted: Font: Times New Roman, 11 pt

Formatted: Justified, Indent: First line: 2 ch, Widow/Orphan control

Formatted: Font: Times New Roman, 11 pt

Formatted: Font: Times New Roman, 11 pt

Formatted: Font: Times New Roman, 11 pt, Bold

Formatted: Font: Times New Roman, 18 pt

Formatted: Justified, Space Before: 0 pt, After: 0 pt

Formatted: Font: Times New Roman, 11 pt

Formatted: Justified, Indent: First line: 2 ch, Widow/Orphan control

Formatted: Font: Times New Roman, 11 pt

Formatted: Font: Times New Roman, 11 pt

Formatted: Font: Times New Roman, 18 pt

Formatted: Line spacing: Double

We ~~would like to~~ thank all medical students who devoted their time to this study.

~~Disclosure~~

The ~~author reports no conflicts of interest in this work.~~ authors thank AiMi Academic Services (www.aimieditor.com) for English language editing and review services.

Author contributions

Wu Zhang, Data Curation, Formal Analysis, Software, Investigation, Writing-Original Draft Preparation, Writing-Review & Editing, Yuquan Huang, Formal Analysis, Software, Visualization, Writing-Original Draft Preparation, Ye Zhou, Supervision, Visualization, Writing-Original Draft Preparation, Jiaojiao Xue, Validation, Writing-Original Draft Preparation, Shan Gao, Supervision, Writing-Original Draft Preparation, Lin Kang, upervision, Validation, Jian Shi, Supervision, Writing-Review & Editing, Tao Zhou, Project Administration, Supervision, Yalong Duan, Formal Analysis, Visualization, Sihan Guo, Validation, Formal Analysis, Writing-Review & Editing, Qingxia Li, Funding Acquisition, Project Administration, Writing-Original Draft Preparation, Writing-Review & Editing

References

1. Sung H, Ferlay J, Siegel RL, Laversanne M, Soerjomataram I, Jemal A, et al. Global Cancer Statistics 2020: GLOBOCAN Estimates of Incidence and Mortality Worldwide for 36 Cancers in 185 Countries. CA Cancer J Clin. 2021;71(3):209-49. ~~Epub 2021/02/05. doi: 10.3322/caac.21660. PubMed PMID: 33538338.~~

2. Kashyap D, Pal D, Sharma R, Garg VK, Goel N, Koundal D, et al. Global Increase in Breast Cancer Incidence: Risk Factors and Preventive Measures. Biomed Res Int.

Formatted: Font: Times New Roman, 11 pt
Formatted: Font: Times New Roman, 11 pt
Formatted: Font: Times New Roman, 11 pt
Formatted: Justified, Indent: First line: 2 ch
Formatted: Font: Times New Roman, 11 pt
Formatted: Font: Times New Roman, 11 pt
Formatted: Justified, Indent: First line: 2 ch, Widow/Orphan control
Formatted: Font: Times New Roman, 18 pt
Formatted: Justified
Formatted: Font: Times New Roman, 11 pt
Formatted: Line spacing: Double

2022;2022:9605439.~~ Epub 2022/04/29. doi: 10.1155/2022/9605439. PubMed PMID: 35480139; PubMed Central PMCID: PMCPMC9038417.~~

3. O'Connor DJ, Davey MG, Barkley LR, Kerin MJ. Differences in sensitivity to neoadjuvant chemotherapy among invasive lobular and ductal carcinoma of the breast and implications on surgery-A systematic review and meta-analysis. ~~Breast (Edinburgh, Scotland). 2022;61:1-10. Epub 2021/12/06. doi: 10.1016/j.breast.2021.11.017. PubMed PMID: 34864494; PubMed Central PMCID: PMCPMC8649952~~Breast. 2022;61:1-10.

4. Thornton MJ, Williamson HV, Westbrook KE, Greenup RA, Plichta JK, Rosenberger LH, et al. Neoadjuvant Endocrine Therapy Versus Neoadjuvant Chemotherapy in Node-Positive Invasive Lobular Carcinoma. ~~Annals of surgical oncology. 2019;26(10):3166-77. Epub 2019/07/26. doi: 10.1245/s10434-019-07564-9. PubMed PMID: 31342392; PubMed Central PMCID: PMCPMC6736696~~Ann Surg Oncol. 2019;26(10):3166-77.

5. Mueller C, Haymond A, Davis JB, Williams A, Espina V. Protein biomarkers for subtyping breast cancer and implications for future research. Expert ~~review of proteomics.~~Rev Proteomics. 2018;15(2):131-52~~. Epub 2017/12/23. doi: 10.1080/14789450.2018.1421071. PubMed PMID: 29271260; PubMed Central PMCID: PMCPMC6104835~~.

6. Dalenc F, Lusque A, De La Motte Rouge T, Pistilli B, Brain E, Pasquier D, et al. Impact of lobular versus ductal histology on overall survival in metastatic breast cancer: a French retrospective multicentre cohort study. ~~European journal of cancer (Oxford, England : 1990). 2022;164:70-9. Epub 2022/02/18. doi: 10.1016/j.ejca.2021.12.031. PubMed PMID: 35176614~~Eur J Cancer. 2022;164:70-9.

~~7~~7. Zhang Z, Kim HJ, Lonjon G, Zhu Y, written on behalf of AMEB-DCTCG. Balance

diagnostics after propensity score matching. Ann Transl Med. 2019;7(1):16.

8. Wang J. To use or not to use propensity score matching? Pharm Stat. 2021;20(1):15-24.

9. Bi G, Liang J, Shan G, Zhan C. Propensity Score Matching for Bias Reduction in Genomic Profiling. Journal of clinical oncology : official journal of the American Society of Clinical Oncology. 2022;40(11):1259-60. Epub 2022/02/22. doi: 10.1200/jco.21.02449. PubMed PMID: 35188823J Clin Oncol. 2022;40(11):1259-60.

810. Morgan CJ. Reducing bias using propensity score matching. Journal of nuclear cardiology : official publication of the American Society of Nuclear Cardiology.J Nucl Cardiol. 2018;25(2):404-6. Epub 2017/08/05. doi: 10.1007/s12350-017-1012-y. PubMed PMID: 28776312.

911. Hossain MJ, Xie L, Caywood EH. Prognostic factors of childhood and adolescent acute myeloid leukemia (AML) survival: evidence from four decades of US population data. Cancer Epidemiol. 2015;39(5):720-6.

12. Mishra P, Pandey CM, Singh U, Gupta A, Sahu C, Keshri A. Descriptive statistics and normality tests for statistical data. Ann Card Anaesth. 2019;22(1):67-72.

13. Damiani G, Bragazzi NL, Garbarino S, Chattu VK, Shapiro CM, Pacifico A, et al. Psoriatic and psoriatic arthritis patients with and without jet-lag: does it matter for disease severity scores? Insights and implications from a pilot, prospective study. Chronobiology international. 2019;36(12):1733-40. Epub 2019/10/28. doi: 10.1080/07420528.2019.1678629. PubMed PMID: 31645138; PubMed Central PMCID: PMCPMC6832868Chronobiol Int. 2019;36(12):1733-40.

1014. Kim GS, Smith AK, Xue F, Michopoulos V, Lori A, Armstrong DL, et al. Methylomic profiles reveal sex-specific differences in leukocyte composition associated with post-traumatic stress disorder. Brain, behavior, and immunity. 2019;81:280-91. Epub 2019/06/23. doi:

Formatted: Line spacing: Double

Formatted: Line spacing: Double

10.1016/j.bbi.2019.06.025. PubMed PMID: 31228611; PubMed Central PMCID: PMCPMC6754791Brain Behav Immun. 2019;81:280-91.

1115. McCloskey RJ, Pei F. The role of parenting stress in mediating the relationship between neighborhood social cohesion and depression and anxiety among mothers of young children in fragile families. Journal of community psychology. 2019;47(4):869-81. Epub 2019/01/23. doi: 10.1002/jcop.22160. PubMed PMID: 30666682; PubMed Central PMCID: PMCPMC6472962J Community Psychol. 2019;47(4):869-81.

1216. Austin PC. The relative ability of different propensity score methods to balance measured covariates between treated and untreated subjects in observational studies. Medical decision making : an international journal of the Society for Medical Decision Making. 2009;29(6):661-77. Epub 2009/08/18. doi: 10.1177/0272989x09341755. PubMed PMID: 19684288Med Decis Making. 2009;29(6):661-77.

13. Foote FW, Jr., Stewart FW. A histologic classification of carcinoma of the breast. Surgery. 1946;19:74-99. Epub 1946/01/01. PubMed PMID: 21022022.

1417. Aslam M. Introducing Kolmogorov-Smirnov Tests under Uncertainty: An Application to Radioactive Data. ACS Omega. 2020;5(1):914-7.

18. Chen X, Li X, Wang J, Zhao L, Peng X, Zhang C, et al. Breast invasive ductal carcinoma diagnosis with a three-miRNA panel in serum. Biomark Med. 2021;15(12):951-963.

19. Danzinger S, Hielscher N, Izso M, Metzler J, Trinkl C, Pfeifer C, et al. Invasive lobular carcinoma: clinicopathological features and subtypes. J Int Med Res. 2021;49(6):3000605211017039.

20. Tan PH, Ellis I, Allison K, Brogi E, Fox SB, Lakhani S, et al. The 2019 World Health Organization classification of tumours of the breast. Histopathology. 2020;77(2):181-5. Epub

Formatted: Line spacing: Double

~~2020/02/15. doi: 10.1111/his.14091. PubMed PMID: 32056259.~~

~~15~~21. Veltman J, Boetes C, van Die L, Bult P, Blickman JG, Barentsz JO. Mammographic detection and staging of invasive lobular carcinoma. ~~Clinical imaging.~~Clin Imaging. 2006;30(2):94-8~~. Epub 2006/02/28. doi: 10.1016/j.clinimag.2005.09.021. PubMed PMID: 16500539~~.

~~16~~22. Siegel RL, Miller KD, Fuchs HE, Jemal A. Cancer Statistics, 2021. CA Cancer J Clin. 2021;71(1):7-33.~~ Epub 2021/01/13. doi: 10.3322/caac.21654. PubMed PMID: 33433946.~~

~~17~~23. Rakha EA, Ellis IO. Lobular breast carcinoma and its variants. ~~Seminars in diagnostic pathology.~~Semin Diagn Pathol. 2010;27(1):49-61.~~ Epub 2010/03/~~

24~~. doi: 10.1053/j.semdp.2009.12.009. PubMed PMID: 20306830.~~

~~18~~. Arpino G, Bardou VJ, Clark GM, Elledge RM. Infiltrating lobular carcinoma of the breast: tumor characteristics and clinical outcome. Breast ~~cancer research : BCR.~~Cancer Res. 2004;6(3):R149-56~~. Epub 2004/04/16. doi: 10.1186/bcr767. PubMed PMID: 15084238; PubMed Central PMCID: PMCPMC400666~~.

~~19~~25. Norgard RJ, Pitarresi JR, Maddipati R, Aiello-Couzo NM, Balli D, Li J, et al. Calcium signaling induces a partial EMT. EMBO Rep. 2021;22(9):e51872.

26. Sokol ES, Feng YX, Jin DX, Basudan A, Lee AV, Atkinson JM, et al. Loss of function of NF1 is a mechanism of acquired resistance to endocrine therapy in lobular breast cancer. ~~Annals of oncology : official journal of the European Society for Medical Oncology. 2019;30(1):115-23. Epub 2018/11/14. doi: 10.1093/annonc/mdy497. PubMed PMID: 30423024; PubMed Central PMCID: PMCPMC6336006~~Ann Oncol. 2019;30(1):115-23.

~~20~~27. Desmedt C, Salgado R, Fornili M, Pruneri G, Van den Eynden G, Zoppoli G, et al. Immune Infiltration in Invasive Lobular Breast Cancer. ~~Journal of the National~~J Natl Cancer ~~Institute~~Inst. 2018;110(7):768-76~~. Epub 2018/02/23. doi: 10.1093/jnci/djx268. PubMed PMID:~~

Formatted: Line spacing: Double

Formatted: Line spacing: Double

~~29471435; PubMed Central PMCID: PMCPMC6037125~~.

~~21~~28. Loo LW, Grove DI, Williams EM, Neal CL, Cousens LA, Schubert EL, et al. Array comparative genomic hybridization analysis of genomic alterations in breast cancer subtypes. Cancer ~~research~~Res. 2004;64(23):8541-9~~. Epub 2004/12/03. doi: 10.1158/0008-5472.Can-04-1992. PubMed PMID: 15574760~~.

~~22~~29. Davis AA, Gerratana L, Clifton K, Medford AJ, Velimirovic M, Hensing WL, et al. Circulating tumour DNA characterisation of invasive lobular carcinoma in patients with metastatic breast cancer. EBioMedicine. 2022;86:104316.~~ Epub 2022/11/05. doi: 10.1016/j.ebiom.2022.104316. PubMed PMID: 36332363; PubMed Central PMCID: PMCPMC9637866.~~

~~23~~30. Wasif N, Maggard MA, Ko CY, Giuliano AE. Invasive lobular vs. ductal breast cancer: a stage-matched comparison of outcomes. ~~Annals of surgical oncology.~~Ann Surg Oncol. 2010;17(7):1862-9~~. Epub 2010/02/18. doi: 10.1245/s10434-010-0953-z. PubMed PMID: 20162457~~.

~~24~~31. Adachi Y, Ishiguro J, Kotani H, Hisada T, Ichikawa M, Gondo N, et al. Comparison of clinical outcomes between luminal invasive ductal carcinoma and luminal invasive lobular carcinoma. ~~BMC cancer. 2016;16:248. Epub 2016/03/27. doi: 10.1186/s12885-016-2275-4. PubMed PMID: 27015895; PubMed Central PMCID: PMCPMC4807554~~BMC Cancer. 2016;16:248.

~~25~~32. P R. Rosen's breast pathology. 3. Philadelphia: Lippincott williams & wilkins. 2009.

Supporting information

S1 Table. IDC group univariate and multivariate Cox regression analysis.~~**Abbreviation:**AAT,Adjuvant Therapy;NSOST,No systemic therapy and/or surgical therapy;PORT,Post-Operative Radiation Therapy;NROS,No radiation and/or cancer-directed surgery.~~

S2 Table. ILC group univariate and multivariate Cox ~~**regression analysis.Abbreviation:**AAT,Adjuvant Therapy;NSOST,No systemic therapy and/or surgical therapy;PORT,Post-Operative Radiation Therapy;NROS,No radiation and/or cancer-directed surgery~~**regressionanalysis.**

Formatted: Font: Times New Roman, 18 pt

Formatted: Justified

Formatted: Font: Times New Roman, 11 pt

Formatted: Font: Times New Roman, 11 pt

Formatted: Font: Times New Roman, 11 pt, Bold

Formatted: Font: Times New Roman, 11 pt, Kern at 16 pt

Formatted: Font: Times New Roman, 11 pt, Kern at 16 pt

Formatted: Justified

Formatted: Font: Times New Roman, 11 pt, Bold, Kern at 16 pt

Formatted: Font: Times New Roman, 11 pt, Not Bold

Formatted: Justified

Dear Editor and Review Experts,

We deeply appreciate your willingness to dedicate your valuable time to reviewing our research. Your professional expertise and precise grasp of details have provided us with invaluable and profound insights, significantly influencing the improvement of our paper. Your feedback and suggestions have not only elevated the quality of our research, but also guided us to think about the issues more comprehensively and rigorously, deepening our research. For this, we express our deep gratitude. In the process of revisiting and revising the paper, we deeply realize the importance of your review in improving our paper. We look forward to your further feedback, and believe that the revised paper will meet your expectations. Once again, we thank you for your thoughtful review and insightful suggestions. We respect and value your opinions, and we hope that our responses and changes meet your expectations.

Below, we have responded in detail to each review comment:

Reviewer 1#

Hi and Thanks for your Email for reviewing the article titled" Delving into Female Breast Cancer: A New Perspective on Survival Differences Between Invasive Lobular and Ductal Carcinomas Revealed by Propensity Score Matching".

1-This is an interesting article with novelty of the result, supporting by:

• Large scale number of cases regardless of different numbers in the clinic (>80 IDC

and <10% ILC, breast cancer)

• Very scientific matching of both pathologic patterns, clinically and biologically and management.

• Novelty of result that confirm the better OS for IDC group regardless our opinion that means the same outcome or even better OS for the ILC group.

• Accepted the same clinical approach to care of the cases with both pathological patterns.

The author's answer： We are extremely grateful for your recognition of our research methods and results, and appreciate your professional review and feedback.

2- In my opinion there is some point of interesting that is missed during discussion and conclusion.

• The EMT is more expected mechanism in the ILC rather than IDC, that may be the reason of more distant metastasis.

• In clinic we use less adjuvant of neoadjuvant chemotherapy in ILC cases comparing to IDC.

• In conclusion this is accepted research with novelty with minor revision in discussion and conclusion.

The author's answer： Thank you very much for the thoughtful evaluation and valuable suggestions you provided for our research.Your observation that epithelial-mesenchymal

transition (EMT) is more common in invasive lobular carcinoma (ILC) than in invasive ductal carcinoma (IDC) is indeed a critical finding. This could be due to the lack of E-cadherin, which stimulates the initiation of EMT and further leads to more common distant metastases in ILC patients. This could also be a key factor causing a poorer disease-specific survival (DSS) in ILC compared to IDC. We have added this important observation to the discussion section and hope that future basic and clinical research can explore the influence of EMT on the prognostic differences between these two pathological types and their underlying mechanisms in greater depth.

As for the influence of clinical treatment strategies on the prognostic differences between ILC and IDC you mentioned, we admit that we did not delve into it in this study. This point has been added to the limitations section of our paper. We look forward to further exploring the causal relationship between treatment methods and prognostic differences among different pathological types in future clinical research.

Once again, thank you for your guidance. We greatly value and will adopt your suggestions. We hope that through revision, the conclusions and discussions of this research will become more rigorous and comprehensive.

Reviewer #2

Breast cancer constitutes a crucial health concern, and investigating this disease is essential to gain deeper insights into its underlying mechanisms, thereby enhancing patient outcomes and

reducing mortality rates. The availability of comprehensive and dependable data sources presents a promising avenue to tackle this challenge, and leveraging cutting-edge data analytical techniques is pivotal to expediting the analysis process and attaining more accurate results. From the title of your manuscript, it appears that your research involves adopting a new perspective on reducing bias caused by confounding variables and accessing large-scale datasets. But there are certain points that need to be addressed:

1.A similar article with the title “Comparison of Overall Survival Between Invasive Lobular Breast Carcinoma and Invasive Ductal Breast Carcinoma: A Propensity Score Matching Study Based on SEER Database” was published in “Frontiers in Oncology” in 2020 (DOI: 10.3389/fonc.2020.590643). This article utilizes the SEER dataset from 2006 to 2016 and employs the propensity score matching (PSM) method for analysis. Therefore, adopting a “new perspective” in the title of your article may not be appropriate, and your research findings could potentially serve as an update to the results presented in the aforementioned article.

The author’s answer： Thank you for your in-depth comments and for drawing our attention to the above-mentioned article. We will reconsider our title based on your suggestions.

2.**On the other hand, it is advisable to refer to the aforementioned article in the introduction of your manuscript and highlight the rationale behind conducting your study, despite the existence of the previous article. In other words, explain the specific need for your research. Furthermore, in the discussion section of your research paper, it**

would be useful to compare your findings with those presented in the previous study.

The author's answer：

Thank you very much for your efforts to ensure the rigor and originality of our research. You mentioned a similar article published in 2020, and we agree with your observation. We believe that although our study has similarities with this article, our research still provides important value and new insights in some key aspects. We will compare our research results with the results of this article and clarify the uniqueness and value of this study. Here are some of our differences:

Data Source: Our research data comes from the SEER Research Plus Data 8 Registries (1978-2019), while the previous study data comes from the SEER 18 tumor registry database (updated to November 2016).

Variable Coverage: Compared to the previous study, we covered a more comprehensive set of covariates for propensity score matching, including race, marital status, primary site, subtype, summary stage, tumor size, and specific types of systemic therapy (such as AAT), the specific sequence of surgery and radiotherapy, etc. These variables undoubtedly have an important impact on the prognosis of breast cancer. Compared with the variables existing in the article, we have made more detailed divisions. The inclusion of these covariates helps us understand the research topic more comprehensively and meticulously. Our Propensity Score Matching (PSM) variables are not subjectively selected, but are relatively objectively screened for variables that significantly affect prognosis through univariate and multivariate Cox regression, thereby ensuring the completeness of the included covariates and avoiding the

unreliability caused by overmatching.

Method selection: Although both our study and the previous one used propensity score matching (PSM), our choice and design of statistical methods are more rigorous and detailed. To ensure the repeatability of the analysis and the reliability of the results, we provided a detailed description of the selection of variables before PSM, the specific method selection of PSM, the control of built-in parameters, and the assessment of the effect after matching (AMD and KS statistics).

Focus on Outcome: Different from the previous study, which focused on OS (Overall Survival), our research focuses on DSS (Disease-Specific Survival). This is because DSS specifically targets deaths caused by the specific disease under study and is a more accurate measure. Our choice of DSS as the main outcome helps us avoid the influence of other potential interfering factors and more accurately reflects the impact of breast cancer on survival. These factors led to our final finding that the DSS of the two groups was statistically significantly different, which might be a potential important reason for the result in that article where IDC patients showed a better OS compared to ILC patients (HR=1.045, P=0.025, 95%CI: 1.007-1.085) in the comparison of OS for unmatched population database. And after matching, it turned out that there was no difference in OS between ILC and IDC patients (HR=1.017, P=0.409, 95%CI: 0.967–1.069).

We have also thoroughly considered citing the aforementioned paper in the introduction section, but we believe it would be more appropriate to cite it in the discussion section. Thus, in accordance with the reviewer's suggestion, we made detailed comparisons between our results and those of the previous study in the discussion section, and we also explained the

reasons for our study. We believe this approach better demonstrates the connection between our research and other studies, and avoids giving readers the impression that our study is specifically targeting that one paper. In fact, our focus is on a class of papers exploring the controversial prognosis differences between IDC and ILC in the academic community. At this point, we would like to express our heartfelt thanks for your professional guidance. Your professional advice and rigorous academic attitude have had a significant impact on our research.

3. Given the large size of your dataset and the sufficient number of outcomes of interest, could you please provide an explanation in your manuscript that clarifies the rationale for using the propensity score method for matching the study participants based on the probability of each individual being assigned to each group?

Since the propensity score matching method failed to correctly match some variables, such as Laterality, would it not be more appropriate to use a conventional survival modeling approach, such as the Cox proportional hazard model, which adjusts for confounding variables in the regression model?

The author’s answer:

Thank you for your valuable questions. In this study, we chose the Propensity Score Matching (PSM) method primarily to reduce selection bias and deal with confounding variables. Despite our dataset's large size and the sufficient number of events, we had to deal with a lot

of potential confounding variables when comparing the DSS of two groups (IDC and ILC). If we directly apply the multivariate Cox proportional hazards model, the complexity of the model might lead to unstable results. However, PSM can reduce selection bias and improve estimation bias caused by confounding variables by matching treatment and control groups in observational studies.

Regarding the issue of matching for Laterality, we didn't include it in PSM because we found no significant correlation between Laterality and DSS in the IDC and ILC groups. This is based on the univariate and multivariate Cox regression analyses we performed. In order to avoid weakening the validity and unnecessary sample loss by including too many irrelevant variables or variables not related to treatment and outcome, we chose not to include Laterality in the matching variables. We will provide additional explanations for this decision in the article.

Additionally, I completely agree with your view of the Cox proportional hazards model. In fact, in the article, we conducted KM analysis on the samples after PSM, as well as Cox proportional hazards regression. For any misunderstandings that may have been caused by a mistake in description, I deeply apologize and have made improvements and additions to the description of the Cox model results in the revised manuscript.

4. Select appropriate keywords based on Mesh in PubMed

The author’s answer:

We sincerely appreciate your comprehensive review and valuable feedback. We have made the necessary modifications to the list of keywords. The revised keywords are now better aligned with MeSH terms and accurately reflect the content of our manuscript.

5. To ensure that your reader understands the meaning of an acronym or abbreviation, provide its complete terminology when you first mention it. For example, when introducing DSS (Disease Specific Survival) in the introduction, provide its full terminology so that the reader understands what it stands for.

The author's answer:

According to your advice, we have made revisions to the paper. When abbreviations or acronyms are mentioned for the first time, we have provided their complete terminology to ensure that readers can understand the meanings of the abbreviations and acronyms.

6. Please display p-values that are less than 0.001 as '< 0.001'.

The author's answer:

Thank you very much for your suggestion. I have followed your advice and now display the results with p-values less than 0.001 as '< 0.001'.

7. Please ensure that percentages are displayed with one decimal place, and p-values are

presented with three decimal places.

The author's answer：

Thank you for your valuable suggestion. I have now adjusted the presentation to ensure that percentages are displayed with one decimal place, and p-values are presented with three decimal places, as per your recommendation. I appreciate your guidance.

Thank you once again for your valuable review and constructive feedback. It has been immensely helpful for our research. We look forward to receiving further feedback from you.

Yours sincerely,

Qingxia Li

Professor, Director

Tel:+86-311-85988779

Email: lqx73@163.com
