# Supplementary material for: Delving into female breast cancer: Distinct disease-specific survival outcomes between invasive lobular and ductal carcinomas revealed by propensity score matching
Source: PLoS One. 2024 Dec 23;19(12):e0300116. doi: 10.1371/journal.pone.0300116 (PMC11665987; doi:10.1371/journal.pone.0300116)
Supplement: Attachment — Submitted filename: Response to Reviewers.docx [file pone.0300116.s006.docx]

Dear Editor and Review Experts,

We sincerely appreciate your valuable time and expertise dedicated to reviewing our research. Your review plays a pivotal role in refining and elevating the quality of our study.We have carefully considered your comments and made corresponding adjustments,anticipating that our responses and modifications align with your expectations. Once again, we thank you for your thoughtful review and insightful suggestions.

Below, we have responded in detail to each review comment:

**Reviewer #3**

The investigation into disease-specific survival (DSS) differences between propensity score-matched invasive ductal carcinoma (IDC) and invasive lobular carcinoma (ILC) patients yielded a significant finding—IDC patients exhibited notably better DSS than their ILC counterparts. Despite the manuscript's well-crafted presentation and thoughtful revisions in response to reviewer feedback, concerns about originality have been raised, given the saturation of similar topics in existing literature. Nevertheless, the study remains valuable and informative for readers.

Here are the requested revisions:

(1) **Typically, breast cancer grades range from I to III. Could the authors clarify the classification of Grade IV?**

**The author’s answer：**We appreciate your thoughtful inquiry regarding the classification of Grade IV in breast cancer grading. As you rightly pointed out, breast cancer grades typically range from I to III, which aligns with the Nottingham or Bloom-Richardson (BR) Score/Grade widely recognized in the field.Additionally, the SEER database documents three distinct systems or formats for describing tumor grading: the 2, 3, or 4 Grade System. The interconversion relationships between these systems can be found on the official SEER database website. In our study, we employ the four-grade system, where Grade IV is defined as "also called undifferentiated or anaplastic.^[1]^ we will ensure a comprehensive explanation of Grade IV in our manuscript to enhance clarity.Your keen observation contributes significantly to the refinement of our work, and we are grateful for your valuable input.

(2) **The estrogen receptor (ER) and progesterone receptor (PR) positive rates in this study appear exceptionally high compared to other reports. Please provide a clear definition of ER and PR positivity in the 'Materials and Methods' section.**

**The author’s answer：**We sincerely appreciate your meticulous observation and professional review. In response to your query, we conducted a thorough analysis of ER and PR positivity rates based on the original data of 510,385 patients from the SEER Research Plus Data 8 Registries, as illustrated in Figure 1. It's important to note that the dataset contained a significant number of Borderline/Unknown and Recode not available entries.To ensure the integrity of our subsequent analyses, we opted to exclude data with unclear ER and PR statuses, as well as other instances of indeterminate clinical characteristics.

As per the SEER database guidelines, ER and PR positivity is defined as follows: If 1% or more cells stain positive, the test results are considered positive;if less than 1% of cells stain positive, the results are considered negative.We have taken your valuable suggestion into consideration and will incorporate this precise definition into the 'Materials and Methods' section of our manuscript to provide transparency and clarity.We express our gratitude for your professionalism and thoroughness in reviewing our work.

|  |  |
| --- | --- |

Figure1: Original distribution of ER and PR status in 510,385 patients with breast cancer.

(3) **In Table 1, 'distant stage' and 'AJCC IV' seem to convey the same concept, yet the numerical values differ. Could the authors explain this disparity?**

**The author’s answer：**Thank you very much for your meticulous observation and insightful feedback. Summary staging is the most basic way of categorizing how far a cancer has spread from its point of origin. Summary staging has also been called General Staging, California Staging, and SEER Staging. Summary Staging uses all information available in the medical record, clinical, and pathological. It is frequently used by tumor registries, but not always understood by physicians.According to the SEER Summary Staging Manual 2000-Breast and Female Genital System (https://seer.cancer.gov/tools/ssm/ssm2000/), "Distant" involves distant sites/lymph nodes, further contiguous extension, and metastasis to specific organs or locations. The description of “Distant” is as follows:

**Distant site(s)/lymph node(s) involved**

**Distant lymph node(s):**

Cervical, NOS

Contralateral/bilateral axillary

Contralateral/bilateral internal mammary (parasternal)

Supraclavicular (transverse cervical)

Other distant lymph node(s)

**Further contiguous extension:**

**Skin over:**

Axilla

Contralateral (opposite) breast

Sternum

Upper abdomen

**Metastasis:**

Adrenal (suprarenal) gland

Bone other than adjacent rib

Contralateral (opposite) breast - if stated as metastatic

Lung

Ovary

Satellite nodule(s) in skin other than primary breast

Upon a thorough examination of our original data and consideration of the aforementioned description, 'distant stage' appears analogous to 'AJCC IV,' yet there are distinctions. Among the 2457 'distant stage' cases, 2353 align with 'AJCC IV,' while an additional 104 cases belong to the AJCC IIIC classification, specifically involving patients with lymph node N3c staging. We sincerely appreciate your attention to detail and the invaluable insights you have provided,and we hope this clarification addresses the observed disparities.Thank you once again for your valuable contributions to our research.

(4) **Given the significance of axillary lymph node (LN) metastasis as a prognostic factor in breast cancer, please include information on axillary LN metastasis in both Table 1 and Table 2.**

**The author’s answer：**Thank you for your valuable advice.I attach great importance to your suggestion regarding the inclusion of axillary LN metastasis. At the data collection stage, we initially considered axillary LN as an independent variable. However, during further analysis, we observed a significant collinear relationship with other factors, which could introduce instability and bias into the results. In order to ensure the accuracy and reliability of the study, we made the decision not to include this variable in the analysis and opted for the comprehensive AJCC stage as a suitable surrogate.

While this particular data did not make it into the final analysis, I am fully cognizant of the pivotal role axillary status plays in breast cancer prognosis.I will continue to follow the research progress in this area and we are collecting local data and taking your suggestion to fully consider axillary status in future studies. Further refine data collection and analysis to validate our results. To ensure the accuracy and reliability of the study.

(5) **I have indicated a minor revision in the manuscript; please incorporate the suggested changes for improved clarity.**

**The author’s answer：**Thank you once more for your professional review. I will enhance the manuscript based on your suggestions to improve its overall clarity.

Yours sincerely,

Qingxia Li

Professor, Director

Tel:+86-311-85988779

Email: lqx73@163.com

1. Liu D, Wu J, Lin C, Andriani L, Ding S, Shen K, Zhu L. Breast Subtypes and Prognosis of Breast Cancer Patients With Initial Bone Metastasis: A Population-Based Study. Front Oncol. 2020 Dec 2;10:580112. doi: 10.3389/fonc.2020.580112. PMID: 33344236; PMCID: PMC7739957.
